# Harnessing the MinION: An example of how to establish long‐read sequencing in a laboratory using challenging plant tissue from *Eucalyptus pauciflora*

**DOI:** 10.1111/1755-0998.12938

**Published:** 2018-10-05

**Authors:** Miriam Schalamun, Ramawatar Nagar, David Kainer, Eleanor Beavan, David Eccles, John P. Rathjen, Robert Lanfear, Benjamin Schwessinger

**Affiliations:** ^1^ Research School of Biology The Australian National University Acton ACT Australia; ^2^ Malaghan Institute of Medical Research Wellington New Zealand; ^3^ Present address: University of Natural Resources and Life Sciences Vienna Austria; ^4^ Present address: Malaghan Institute of Medical Research Wellington New Zealand

**Keywords:** genomics, high‐molecular‐weight DNA, long‐read sequencing, nanopore

## Abstract

Long‐read sequencing technologies are transforming our ability to assemble highly complex genomes. Realizing their full potential is critically reliant on extracting high‐quality, high‐molecular‐weight (HMW) DNA from the organisms of interest. This is especially the case for the portable MinION sequencer which enables all laboratories to undertake their own genome sequencing projects, due to its low entry cost and minimal spatial footprint. One challenge of the MinION is that each group has to independently establish effective protocols for using the instrument, which can be time‐consuming and costly. Here, we present a workflow and protocols that enabled us to establish MinION sequencing in our own laboratories, based on optimizing DNA extraction from a challenging plant tissue as a case study. Following the workflow illustrated, we were able to reliably and repeatedly obtain >6.5 Gb of long‐read sequencing data with a mean read length of 13 kb and an N50 of 26 kb. Our protocols are open source and can be performed in any laboratory without special equipment. We also illustrate some more elaborate workflows which can increase mean and average read lengths if this is desired. We envision that our workflow for establishing MinION sequencing, including the illustration of potential pitfalls and suggestions of how to adapt it to other tissue types, will be useful to others who plan to establish long‐read sequencing in their own laboratories.

## 1 INTRODUCTION

Single‐molecule nanopore sequencing records changes in electrical current as individual tagged DNA molecules pass through an engineered pore across a chemical gradient (Jain, Olsen, Paten, & Akeson, 2016). Groups of consecutive bases cause a characteristic shift in current, and this can be deconvoluted to infer the individual base sequence of the DNA molecule, a process referred to as basecalling. This technology can sequence DNA fragments of varied lengths, from a few hundred bases to over a megabase (Mb), which compares favourably to sequencing by synthesis (e.g., Illumina), which is limited to hundreds of bases (Leggett & Clark, 2017). Long reads have a number of important applications, including improving the accuracy and efficiency of genome assembly, especially for genomes that contain long low‐complexity regions; detailed investigation of segmental duplications and structural variation (Jain et al., 2018); major histocompatibility complex (MHC) typing (Liu et al., 2017); and detecting methylation patterns (Simpson et al., 2017). The number of genome assemblies using nanopore data either exclusively or in combination with other sequencing data is steadily increasing, for example the 3.5 gigabase (Gb) human genome, the 860 Mb European eel genome, the 1 Gb genome of the wild tomato species *Solanum pennellii *and the 135 Mb genome of *Arabidopsis thaliana *(Jain et al., 2018; Jansen et al., 2017; Michael et al., 2018; Schmidt et al., 2017). In short, nanopore sequencing solves the technical challenges of reading long DNA fragments, while still having room for improvement in terms of per read accuracy.

The Oxford Nanopore Technologies (ONT) MinION makes long‐read sequencing accessible to most laboratories outside of a dedicated genome facility. It has very low capital cost, has the potential to generate more than 1 Gb of sequence data per 100 USD, has a footprint about the size of an office stapler and runs on a standard desktop or laptop computer. The MinION uses small consumable flowcells for sequencing, which contain fluid channels that flow samples onto a sequencing matrix and provide a small amount of fluid waste storage.

This democratization of sequencing brings the challenge that every laboratory has to establish the sequencing platform and concomitantly, new DNA extraction and library preparation protocols. One of the primary remaining challenges is to extract and purify very long DNA fragments from the organisms or tissues of interest. This is especially important for nanopore sequencing as the native DNA molecules are directly translocated through the nanopore. Any contaminants and impurities directly interfere with the optimal sequencing outcome. Acquiring some data is easy, but it can be challenging and time‐consuming to obtain reliable and good yields (>5 Gb as of writing of this article 03/2018) from challenging starting material. Here, we illustrate the workflow we applied to establish MinION sequencing in our laboratories using the tree species *Eucalyptus pauciflora* as a case study. It is challenging to extract high‐purity and high‐molecular‐weight DNA from E. pauciflora because the mature leaf tissue is physically tough, and because it contains very high levels of secondary metabolites which are known to reduce the efficacy of DNA extraction protocols (Coppen, 2002; Healey, Furtado, Cooper, & Henry, 2014). We illustrate reliable and repeatable ways of measuring DNA purity to optimize output from the MinION sequencer. We discuss important considerations for DNA library preparation, and methods to control and optimize the final distribution of read lengths. We show that during DNA extraction, small alterations in sample homogenization protocols can drastically alter DNA fragment lengths; introduce a novel low‐tech size selection protocol based on solid‐phase reversible immobilization (SPRI) beads; and assess the impact of size selection via electrophoresis and controlled mechanical DNA shearing. Finally, we introduce an open‐source MinION user group that shares DNA extraction, size‐selection and library preparation protocols for many additional organisms, making our workflow applicable well beyond the case study presented here.

## 2 METHODS

### 2.1 Tissue collection

*Eucalyptus pauciflora* leaf tissue was collected from Thredbo, New South Wales (NSW), Australia. After harvesting, the young twigs were transported in plastic bags and stored in darkness at 4°C in water until DNA extraction.

### 2.2 High‐molecular‐weight DNA extraction and clean up

We extracted high‐molecular‐weight DNA based on Mayjonade's DNA extraction protocol optimized for our eucalyptus samples (Mayjonade et al., 2016; Schalamun & Schwessinger, 2017b) (Supporting information Appendix S1). Each extraction was carried out with 800–1,000 mg leaf tissue which was cut into small pieces and split between 8 separate 2‐ml Eppendorf tubes, each containing two metal beads of 5 mm in diameter, before freezing in liquid nitrogen. We lysed the tissue mechanically by grinding using the Qiagen TissueLyser II for 35 s at 25 Hz. Pulverized tissue was suspended in 700 µl SDS lysis buffer (1% w/v PVP40, 1% w/v PVP10, 500 mM NaCl, 100 mM Tris–HCl pH 8.0, 50 mM EDTA, 1.25% w/v SDS, 1% w/v sodium metabisulfite, 5 mM DTT, Milli‐Q water and heated to 64°C for 30 min to inactivated DNases. One µL RNase A (10 mg/ml) (Thermo Fisher) per 1 ml lysis buffer was added to the mixture after the heat treatment, followed by incubation at 37°C for 50 min at 400 rpm on a thermomixer. Twenty minutes into the incubation we added 10 µl Proteinase K (800 Units/ml) (NEB). To precipitate proteins, the tubes were cooled on ice for 2 min before adding 0.3 vol (210 µl) 5 M potassium acetate pH 7.5 and mixed by inverting the tube 20 times. The precipitates containing leaf material and proteins were removed by centrifugation at 8,000 *g* for 12 min at 4°C. We transferred the supernatants to new tubes and added 1.0 vol (V/V) of the SPRI beads solution as described below followed by an incubated on a hula mixer for 10 min. After a brief (pulse) centrifugation step in a microcentrifuge, we placed the tube in a magnetic stand so that the beads bound to the rear of the tube, allowing for removal of the supernatant. We then washed the beads twice with 1 ml 70% ethanol. For the wash, we kept the tube on the magnetic stand throughout the wash procedure to avoid loss of DNA bound to the beads (the tube can be rotated 360° within the stand, allowing comprehensive washing while ensuring bead retention). After the second wash, we centrifuged the tube briefly again to remove the last traces of ethanol. The beads were dried for no longer than 30 s before elution of the DNA in 50 µl TE buffer preheated to 50°C, for 10 min.

We further purified the samples using a chloroform: isoamylalcohol extraction. The eight aqueous DNA solutions were pooled to a total of 400 µl to which one volume of chloroform:isoamylalcohol (24:1) was added, mixing by inversion for 5 min. The phases were separated by centrifugation at 5,000 *g* for 2 min at room temperature (RT). We transferred the upper, DNA containing phase to a fresh tube performing another round of the chloroform:isoamylalcohol purification. After the second extraction, the DNA was precipitated by adding 0.1 volume 3 M sodium acetate pH 5.3 and 1 volume 100% cold ethanol, followed by centrifugation at 5,000 *g* for 2 min at RT. The short centrifugation at low speed may reduce DNA yields but potentially precipitates longer fragments in favour of shorter fragments. The transparent pellet was washed with 70% ethanol and resuspended in 50 µl 10 mM Tris–HCl pH 8.0 for 2 hr at room temperature. The solubilized DNA was stored at 4°C until library preparation, for a maximum of 10 days.

### 2.3 DNA size selection

#### 2.3.1 g‐TUBE shearing

We processed 5 µg of pure HMW DNA through a g‐TUBE (Covaris) in an Eppendorf 5,418 centrifuge at 3,800 rpm (1,243 *g*), which is slightly lower than the recommended 4,200 rpm (1519 *g*) for 20 kb fragment size shearing, for a total of 2 min.

#### 2.3.2 BluePippin size selection

We used the BluePippin system (Sage science) with 0.75% dye‐free Agarose cassettes and marker S1, selecting for fragments >20 kb using 6 µg sample for each lane and not the recommended 5 µg, because we were expecting a higher recovery from a slightly higher input amount as suggested by the sales representative.

### 2.4 Removal of small DNA fragments <1.5 kb with optimized SPRI beads

In order to purify and remove small fragments from our DNA samples, we optimized a SPRI beads solution which we used for clean ups and library preparations. The improved beads solution consist of 11% PEG 8,000, 1.6 M NaCl, 10 mM Tris–HCl pH 9.0, 1 mM EDTA, 0.4% Sera‐Mag SpeedBeads (GE Healthcare PN 65152105050250) (Schalamun & Schwessinger, 2017a) (Supporting information Appendix S2). For the clean‐up procedure, 0.8 vol of this beads solution was mixed with the DNA sample and incubated on a hula mixer for 10 min. After a brief (pulse) centrifugation step in a microcentrifuge, we placed the tube in a magnetic stand so that the beads bound to the rear of the tube, allowing for removal of the supernatant. We then washed the beads twice with 1 ml 70% ethanol for all steps except after the last adapter ligation step. For the last postadapter ligation step, SPRI beads were washed with ONT's recommended ABB solution instead of Ethanol. For the wash, we kept the tube on the magnetic stand throughout the wash procedure to avoid loss of DNA bound to the beads (the tube can be rotated 360° within the stand, allowing comprehensive washing while ensuring bead retention). After the second wash, we centrifuged the tube briefly again to remove the last traces of ethanol. The beads were dried for no longer than 30 s before elution of the DNA in 50 µl Tris–HCl pH 8.0 preheated to 50°C, for 10 min.

### 2.5 DNA quality control

DNA concentrations were determined using the Qubit dsDNA BR (Broad Range) assay kit (ThermoFisher). The purity of the sample was measured with the NanoDrop, assessing curve shape, the 260/280 nm and 260/230 nm values, and congruence of concentrations with the Qubit values. The DNA was examined after 0.8% agarose gel electrophoresis containing 0.001% (V/V) SYBR Safe dye (ThermoFisher) in 1X TBE buffer (10.8 g/L Tris base (10 mM), 5.5 g/L boric acid, 0.75 g/L EDTA, pH 8.3) for 45 min at 100 V. For higher resolution, pulsed‐field gel electrophoresis (PFGE) was used with a 1.5% agarose gel in 0.5X TBE running buffer, run for 17.7 hr at 6 V/cm and 1.4 s initial and 13.5 s final switch time. The gel was stained after the electrophoresis with 5 µl SYBR Safe dye in approximately 200 ml Milli‐Q water.

### 2.6 Library preparation and sequencing

We followed two versions of the 1D SQK‐LSK108 ligation protocol; mostly, we used the SQK‐LSK108 selecting for long reads (SQK‐LSK108long) and in some cases the regular SQK‐LSK108 protocol (Supporting information Table S1). In the following values for SQK‐LSK108long are shown without brackets and values for the SQK‐LSK108long in square brackets []. We started the FFPE repair step with ~4 µg [~1.5 µg] HMW DNA dissolved in 46 µl Tris–HCl pH 8.0. Therefore, the sample was incubated at 20°C for 15 min with 5 µl [2 µl] FFPE repair mix (NEB) and 16.3 µl [6.5 µl] FFPE repair buffer (NEB), filling up with 87.7 µl [8.5 µl] nuclease free water (NFW) to 155 µl [62 µl] reaction volume. For the reactions following clean‐up steps, instead of the recommended AMPure XP beads we used 0.8 vol (V/V) of our optimized SPRI beads solution, washing twice with 70% EtOH (Schalamun & Schwessinger, 2017a). We then incubated the resultant DNA eluted in 100 µl [45 µl] NFW with 6 µl [3 µl] Ultra II End‐Prep enzyme mix (NEB) and 14 µl [7 µl] Ultra II End‐Prep buffer (NEB), totalling 120 µl [60 µl] reaction volume, for 5 min at 20°C followed by 5 min at 65°C. The DNA was again cleaned with 0.8 vol (V/V) SPRI beads solution, washing with 70% EtOH. For the adapter ligation, we added 50 µl [50 µl] Blunt/TA Master mix (NEB) and 20 µl [20 µl] AMX Adapter mix (ONT) to the 30 µl [30 µl] end‐repaired DNA and incubated for 10–15 min at RT. For this SPRI beads step, we used ONTs ABB instead of EtOH for the wash and eluted the DNA in 13 µl [13 µl] elution buffer (ELB) (ONT). For sequencing, the final DNA was mixed with 35 µl [35 µl] RBF (ONT), 25.5 µl [25.5 µl] LLB (ONT) and 2.5 µl [2.5 µl] NFW, totalling 75 µl DNA in solution to be loaded onto the flowcell. Before loading, the flowcell was primed with a solution consisting of 480 µl RBF and 520 µl NFW, and first 800 µl of this solution was added into the sample port with a closed SpotON port, incubating for 5 min followed by the remaining 200 µl but with an open SpotON port. After the priming was completed, we added the prepared DNA drop‐by‐drop into the open SpotON port. The sequencing software minknow version 1.7.3 was installed on a computer with minimum of 4 cores running a Linux operating system (Ubuntu 14.4).

## 3 RESULTS

### 3.1 Optimizing sequencing output

#### 3.1.1 DNA sample purity

The first goal of our project was to optimize extraction protocols to yield highly intact and high‐purity DNA suitable for long‐read sequencing. High purity of DNA is defined by Nanodrop spectrophotometer (Thermo Fisher) absorbance of DNA with a 260/280 nm ratio between 1.8 to 2.0 and a 260/230 nm ratio between 2 and 2.2 when all absorbance at 260 nm is due to double‐stranded (ds) DNA (Desjardins & Conklin, 2010; Mackey & Chomczynski, 1997). Therefore, it is important that the ratio of DNA concentrations measured on the Qubit and Nanodrop instruments, respectively, should be at least 1:1.5 and optimally 1:1. A 1:1 ratio indicates that most DNA molecules are double‐stranded and that no other molecules (e.g., RNA) are present that absorb at 260 nm (e.g., Qubit: 100 ng/µl; Nanodrop: 150 ng/µl gives an acceptable ratio of 1:1.5 (O'Neill, McPartlin, Arthure, Riedel, & McMillan, 2011)).

**Figure 1:**
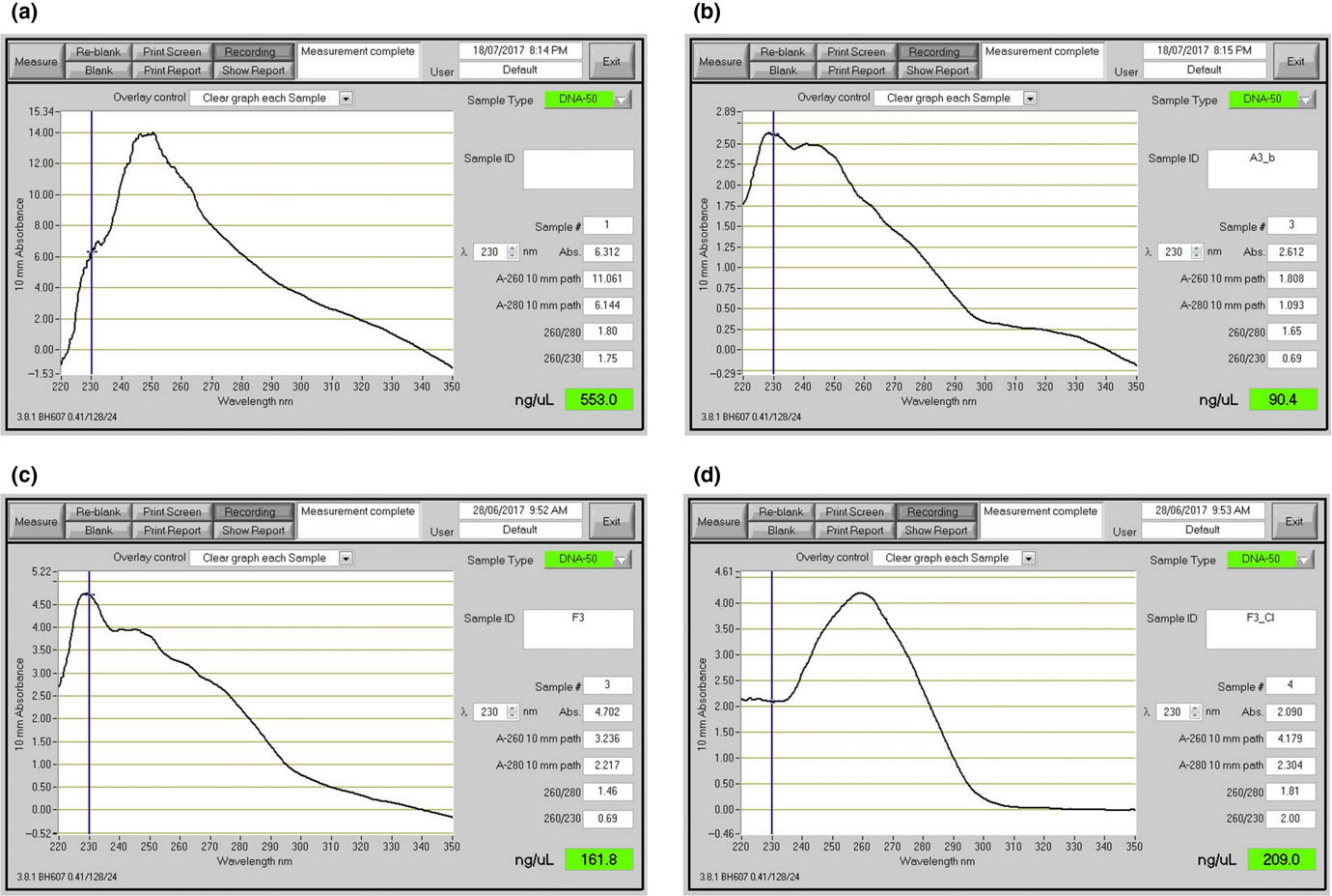
Illustration of different purity DNA preparations. Nanodrop readings of different DNA preparations. (a) DNA extraction with CTAB lysis buffer followed by phenol: chloroform: isoamylalcohol extraction (Schwessinger & Rathjen, 2017). (b) Sample A after SPRI beads clean up. (c) DNA extraction using SDS lysis buffer and SPRI beads purification (Mayjonade et al., 2016). (d) Sample C followed by an additional chloroform: isoamylalcohol purification step. The curves are representations of 260/280 and 260/230 quality control numbers which can be found in Supporting information Table S1 [Colour figure can be viewed at wileyonlinelibrary.com]

In our workflow, we first aimed to recover high‐molecular‐weight DNA with a Nanodrop/Qubit concentration ratio that was close to 1:1. We then optimized DNA purity based on 260/280 nm ratios, which are indicative of protein contamination, and 260/230 nm ratios, which are indicative of contamination by salts, phenol and carbohydrates (O'Neill et al., 2011). To achieve this, we first tested a well‐established hexadecyltrimethylammonium bromide (CTAB) extraction protocol to extract DNA from *E. pauciflora* leaves collected in June 2017 from adult trees in the Kosciuszko National park near Thredbo, New South Wales, Australia (Doyle & Doyle, 1987, 1990 ; Healey et al., 2014; Schwessinger & Rathjen, 2017). While the CTAB protocol returned good yields of double‐stranded DNA (~5 µg DNA per g tissue), the Qubit/Nanodrop ratio of 0.05 indicated significant contamination with RNA or single‐stranded DNA. Nanodrop absorption spectra from 220 to 350 nm (Figure 1a) deviated drastically from pure DNA absorption curves, revealing the presence of contaminants (Figure 1d). In such cases, it is often recommended to clean the DNA using SPRI paramagnetic beads in combination with a polyethylene glycol (PEG) and sodium chloride (NaCl) mixture, such as AMPure XP beads (Beckman Coulter). These beads bind to the DNA, but most contaminants do not and can be washed away (Krinitsina, Sizova, Zaika, Speranskaya, & Sukhorukov, 2015; Mayjonade et al., 2016). We were able to improve sample quality slightly in terms of Qubit to Nanodrop concentration ratios by adding the standard measure of 0.45 vol (V/V) AMPure XP beads (Figure 1b) but repeating this step did not increase the purity of the DNA further as measured by 260/230 nm and 260/280 nm ratios. Next, we tested an extraction method employing the detergent sodium dodecyl sulphate (SDS) which contains a PEG‐NaCl precipitation step to capture the DNA onto SPRI beads. This approach has been reported to work well with many species including sunflower, human, and *Escherichia coli* (Mayjonade et al., 2016). Using this approach, we recovered high levels of double‐stranded DNA (Qubit/Nanodrop = 1:1.5), but the Nanodrop absorption curves still indicated the presence of contaminants in the final DNA extract (Figure 1c). Again, we were unable to improve the DNA purity by repeated SPRI clean up steps, obtaining a maximum of 1.5 for the 260/280 nm and a maximum of 1.0 for 260/230 nm ratios. As an alternative method, we cleaned the crude DNA obtained from the SDS‐based method using a chloroform: isoamylalcohol extraction followed by isopropanol or ethanol precipitation of the DNA, as described for some fungal DNA samples (Dong, 2017). This consistently resulted in high‐purity DNA with Qubit/Nanodrop ratios of 1:1–1.5, 260/280 nm ratios of ~1.8, 260/230 nm ratios of ~2.0 and excellent Nanodrop absorbance curves (Figure 1d).

ONT 1D (1D because only one DNA strand is sequenced) library preparations involve the ligation of sequencing adapters at both 3’ ends of end‐repaired double‐stranded DNA. Sequencing adapters carry a motor protein that guides the DNA to the pore and regulates the translocation speed of the DNA across the pore. In addition, they carry a characteristic DNA sequence which is used by basecallers to recognize the translocation start of a new DNA molecule (Jain et al., 2016; Leggett & Clark, 2017). We tested the effect of sample impurities on MinION output using the 1D ligation protocol. Our three samples differed primarily in their 260/230 nm ratios. One suboptimal sample (sample 5, Table 1), for which no chloroform: isoamylalcohol clean up step was performed, had a low ratio of 1.0. The other two samples (samples 10 and 27, Table 1) had close‐to‐optimal ratios of 2.1 and 2.3, respectively. The sample with the low 260/230 nm ratio yielded an order of magnitude less sequence data from a single flowcell compared to the other two samples (0.7 Gb vs. ~7 Gb, respectively, Table 1, Supporting information Table S1). It seems likely that the contaminants causing the reduced 260/230 nm ratio inhibited the library preparation or the sequencing itself.

**Table 1:** DNA purity impacts sequencing yields

Sample	Qubit (ng/µl)	Nanodrop (ng/µl)	260/280	260/230	Yield (Gb)	Yield_Q7 _(Gb)
10	178	203	1.8	2.1	6.0	5.9
27	142	188	1.8	2.3	7.8	7.4
5	57	80	1.7	1.0	0.7	0.7

Note

Comparison of yield per flowcell for different quality samples. Impact of sample quality measured by 260/280 and 260/230 nm ratios (Nanodrop data) on the final sequence output measured in Gb per flowcell (Figure 1). Sample #10 and #27 are two representative sequencing runs. #5 is a run with low input DNA purity.

#### 3.1.2 Sequencing library preparation

**Figure 2:**
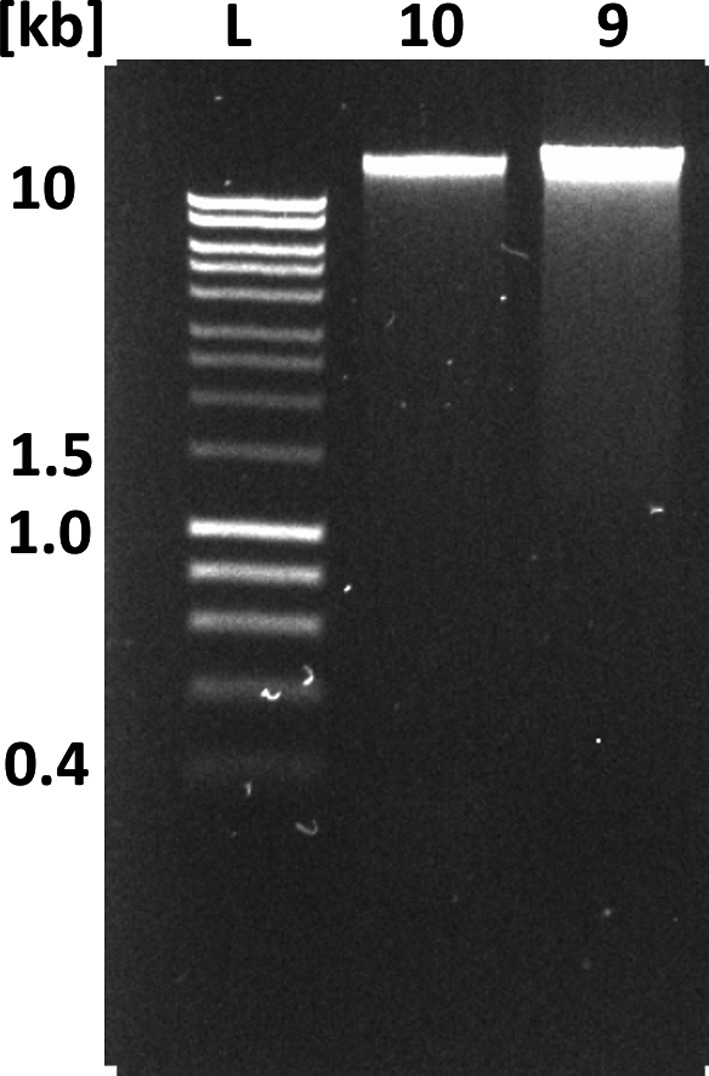
Illustration of the impact on DNA extraction procedures on DNA fragment length. 0.8% agarose gel of 100 ng DNA prepared with two different DNA extraction procedures as explained in the main text. Lane #1 (L) HyperLadder 1 kb (Bioline). #2 (sample 10) DNA extracted following the default HMW DNA extraction protocol with mean read length of 13 kb as shown in Table 2. #3 (sample 9) DNA accidentally sheared during the extraction procedure with mean read length of 5 kb as shown in Table 2

**Figure 3:**
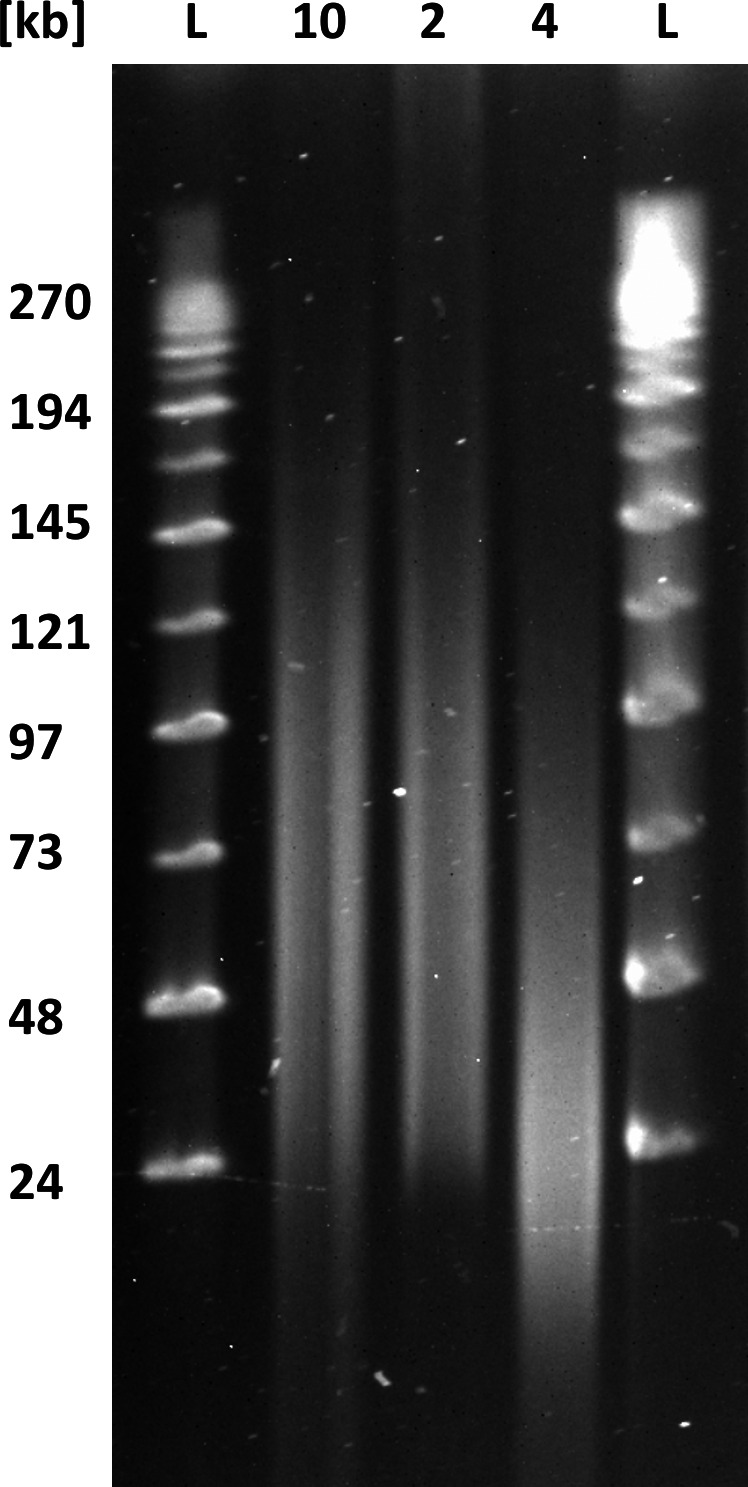
Purposeful mechanical shearing and high‐pass filtering alter DNA fragment length distribution. Pulsed‐field gel electrophoresis of differently treated DNA samples. Lane #1 and #5 (L) MidRange II PFG marker (BioLabs). Lane #2 (sample 10) DNA extracted following the default HMW DNA extraction protocol (mean read length of 13 kb as shown in Table 4). Lane #3 (sample 2) same DNA extraction as in #2 followed by size selection with the BluePippin using 20 kb high‐pass filtering (a mean read length of 26 kb as shown in Table 4). Lane #4 (sample 4) same DNA extraction as in #2 followed by mechanical shearing with a g‐TUBE (a mean read length of 11.8 kb as shown in Table 3)

The manufacturer‐recommended kit for library preparation, which was LSK108 at the time of the experiments, involves DNA repair and end‐prep and is optimized for 0.2 pmol of input DNA with an average fragment size of 8 kb, which in turn requires 1 µg of double‐stranded DNA. This implies that the DNA input as expressed in mass needs to be adjusted according to the concentration of free DNA ends available for adapter ligation, which is a function of fragment length (Mayjonade, 2018; Schwessinger, 2018). The molarity of the DNA sample can be calculated using the Promega BioMath calculator (https://www.promega.com/a/apps/biomath/) which requires the average fragment length to calculate the respective DNA mass for 0.2 pmol. For example, 0.2 pmol of DNA of mean length 24 kb requires a DNA input of 3 µg. In our case, we estimated a mean DNA fragment length of ~30 kb based on the slight low‐molecular‐weight smear observed during 0.8% agarose gel electrophoresis (Figure 2) and the strongest staining between 24 and 97 kb during pulsed‐field gel electrophoresis (PFGE) which provides higher‐resolution in the high‐molecular‐weight range (Figure 3). When estimating mean DNA fragment length based on fluorescent intensity (e.g., after staining with SYBR red or ethidium bromide), it is important to consider that smaller DNA molecules incorporate less dye so appear fainter during imaging. For example, even faint DNA smears below 10 kb can indicate the significant presence of short DNA fragments that are best avoided if long‐read lengths are a primary goal of the sequencing effort (see below.). Failure to account for this can easily lead to overestimation of mean DNA fragment length, and miscalculation of the true molar concentration of DNA fragments.

**Figure 4:**
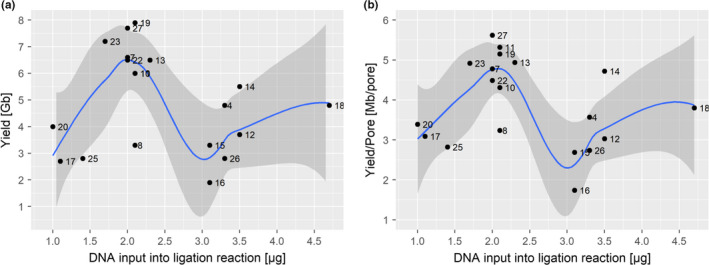
Optimized DNA input into the sequencing adapter ligation reaction. DNA input [µg] into the adapter ligation reaction of the 1D library preparation (*x*‐axis) versus final sequence yields [Gb] (A) or versus sequencing yield normalized by available pores during flowcell QC [Mb/pore]. The points in both graphs are labelled by the sample number (Supporting information Table S1), with higher numbers representing runs with more experience. Graphs also show a locally smoothed regression curve with 95% confidence intervals [Colour figure can be viewed at wileyonlinelibrary.com]

As a starting point, we defined the optimal DNA input based on our initial mean fragment length estimate of 30 kb. This was followed by empirical adjustments from plotting sequencing outputs versus the DNA input into adapter ligation (Figure 4). This approach revealed an optimum of ~2 µg dsDNA (Figure 4), which required an input of 2.9 µg DNA for the DNA preparation stage considering typical losses of 30% after clean up using in‐house SPRI beads (see below). Neither decreasing or increasing the DNA input improved the sequencing output, due to too few adapter‐DNA molecules, or too many free DNA molecules potentially interfering with the sequencing reaction. Assuming that 2.9 µg input DNA was the equivalent of 0.2 pmol (recommended concentration as per the ONT protocol LSK108 that was used), we estimate a mean DNA fragment length of 23 kb for our sample preparation. This suggests we initially overestimated the mean DNA fragment length and highlights the difficulty of estimating these values based on gel imaging.

### 3.2 Altering DNA fragment length and DNA read length

Several factors influence DNA stability during extraction, including chemical properties of the buffer and the physical forces applied during tissue homogenization, phase separation and pipetting (Klingstrom, Bongcam‐Rudloff, & Pettersson, 2018). The buffer composition is the least flexible factor, especially for difficult tissues such as field samples of eucalyptus leaves that require complex buffers for DNA extraction (see above). In contrast, the conditions during tissue homogenization can be adjusted more easily by changing treatment type and length. Optimizing these parameters is very important when optimizing DNA fragment length.

To demonstrate the effect of superficially minimal changes in sample handling, we compared DNA fragment length with sequencing read lengths between two sets of samples that were subjected to different tissue homogenization procedures. Our standard tissue homogenization method for eucalyptus leaves consisted of crushing frozen samples for 35 s with two 5‐mm metal beads in a Qiagen TissueLyser II at 24 Hz. We established 35 s as the best treatment time in terms of DNA yield and DNA integrity when testing a series of treatment times ranging between 20 and 120 s. To maintain the frozen state, each Eppendorf tube as well as the grinding rack was frozen in liquid nitrogen before the homogenization step. In an attempt to improve throughput, we tested the effect of homogenizing samples in larger batches, which likely led to a situation where not all samples were completely frozen throughout the procedure while still being cooled. This change in handling clearly impacted the DNA fragment length distribution as estimated by 0.8% agarose gel electrophoresis. DNA samples extracted using our standard method migrated largely as a single high‐molecular‐weight DNA band at the upper limit of resolution (~23 kb) and well above the 10 kb size standard. For this sample, we observed only a light smear visible to 2.5 kb. In contrast, the tissue sample treated in larger batches showed an enhanced low‐molecular‐weight smear visible to 1 kb (Figure 2) in addition to the large HMW band. This suggests that the average DNA fragment length was reduced in this sample. To more accurately assess the effect of the change in tissue handling, we ran the second DNA extraction on a single flowcell and compared the results to those of two flowcells loaded with DNA prepared using the standard (constantly frozen) tissue handling method. The relatively subtle increase in visible DNA smearing on the agarose gel (Figure 2) belied a drastic shift in read‐length distributions; the mean read length dropped from ~13 kb to 4.9 kb and the median from ~7 to 2.5 kb (Table 2). This illustrates that even a slight change in DNA smearing can have a huge impact on sequencing output.

**Table 2:** DNA integrity impacts sequencing read length

Sample	Shearing	N50_Q7_ (kb)	Mean_Q7 _(kb)	Median_Q7 _(kb)	Yield (Gb)	Yield_Q7 _(Gb)
10	NO	25.8	12.4	6.2	6.0	5.9
27	NO	26	13.2	7.5	7.8	7.4
9	Sheared during extraction	9.2	4.9	2.5	3.5	3.5

Note

Read‐length comparison for samples sheared during the extraction process. Comparison of N50_Q7, _mean read length_Q7 _and median read length_Q7_ between untreated samples (#10 and #27) and the DNA sample sheared during DNA extraction as shown in Figures 2 and 3 (#9).

**Figure 5:**
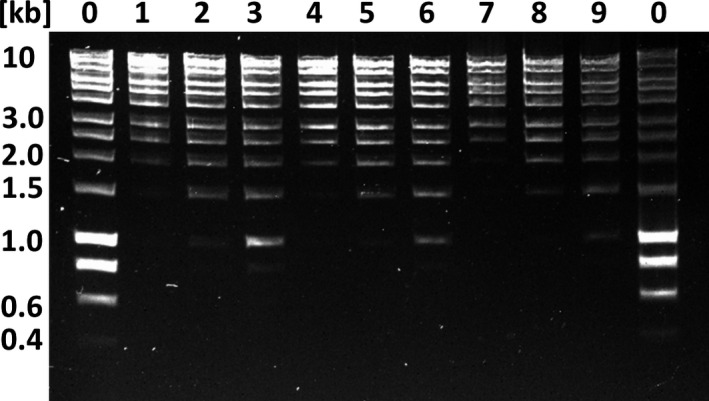
Improved DNA size selection using an adapted PEG‐NaCl‐SPRI beads protocol. Each lane represents 150 ng DNA before size selection. Lanes 0 contain the HyperLadder 1 kb (BioLine) as untreated control. Lanes 1–3 are DNA ladder size selected with 0.4 vol, 0.45 vol, and 0.5 vol (V/V) Agencourt AMPure XP beads. Lanes 4–6 are DNA ladder size selected with 0.8 vol, 0.9 vol, 1.0 vol (V/V) of the adapted PEG‐NaCl‐SPRI beads solution without Tween 20 (Schalamun & Schwessinger, 2017a). Lanes 7–9 are DNA ladder size selected with 0.8 vol, 0.9 vol, 1.0 vol (V/V) of the adapted PEG‐NaCl‐SPRI beads solution with 0.25% Tween‐20 (Nagar & Schwessinger, 2018a)

Because our focus for this project was on generating reads >5 kb to assemble a repeat‐rich genome de novo, we reasoned that it would be beneficial to depleted smaller DNA fragments (<1–2 kb) from all samples. AMPure XP beads can be used to size‐select DNA fragments in the range 100–500 bp (He, Zhu, & Gu, 2013; Schmitz & Riesner, 2006). However, it is not possible to remove DNA fragments larger than ~1,000 bp with an 0.45 vol (V/V) of AMPure XP beads (Figure 5). While some protocols recommend 0.4 vol (V/V) for size selection (Figure 5), these low AMPure XP volumes often failed to recover significant amounts of DNA for most more complex sample types in our hands. This is likely caused by the fact that 0.4 vol (V/V) AMPure XP bead solution is very close to the sigmoidal threshold that causes the NaCl concentration to fall below 0.4 M, leading to complete sample loss at the given PEG concentration of 8.2% (V) (He et al., 2013). We reasoned that by adjusting the PEG and NaCl concentrations, which precipitate DNA in a cooperative manner, we might be able to select a higher average DNA fragment length and thereby remove unwanted smaller DNA fragments while still being able to recover significant amount of input DNA (Lis & Schleif, 1975; Ramos, de Vries, & Ruggiero Neto, 2005). Using 0.8 vol (V/V) of our adjusted SPRI beads mixture (which translates to final PEG concentrations of 4.8% (V) and 0.7 M NaCl), we depleted DNA fragments of up to 1.5 kb (Figure 5) (Schalamun & Schwessinger, 2017a), which we later further improved slightly in terms of size selection and sample handling by avoiding DNA clumping at high concentrations (>100 ng/µl) when adding 0.25% Tween‐20 (V/V) (Figure 5) (Nagar & Schwessinger, 2018a). We used this adapted SPRI beads mixture subsequently, without Tween‐20 at the time of sequencing, for DNA sample clean up and during library preparation.

**Figure 6:**
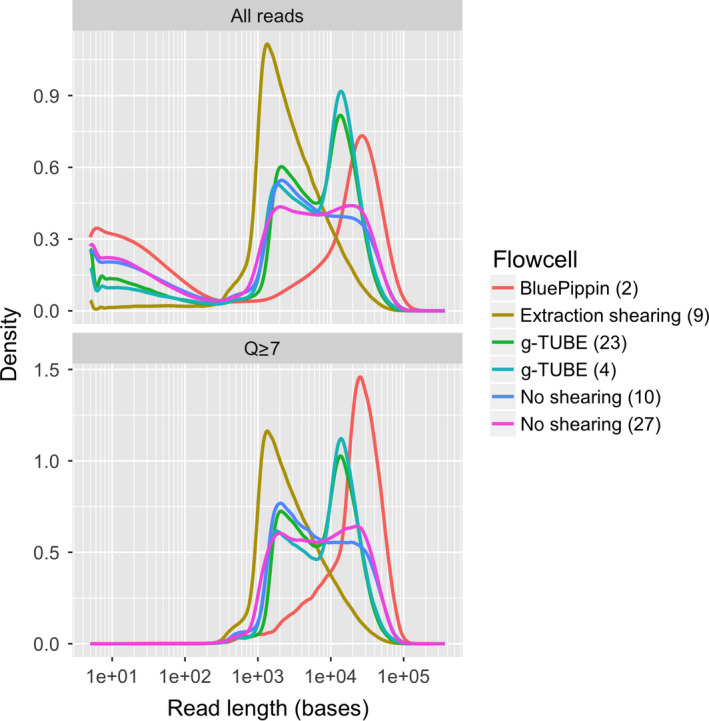
The impact of DNA extraction protocol on the distribution of read lengths from ONT sequencing. Each line represents the read‐length distribution for a single flowcell. The *x*‐axis shows the read lengths on a log scale, and the *y*‐axis shows the density of reads at a particular length. The top panel shows data for all reads, and the bottom panel shows the same data, but with reads that have a mean quality (Q) score <7 removed [Colour figure can be viewed at wileyonlinelibrary.com]

We next assessed the effect of DNA shearing and gel‐based size‐selection procedures on sequencing throughput and read‐length distribution. In the case of DNA shearing, our hypothesis was that a more unimodal size distribution of shorter DNA fragments with a peak of about 20 kb (Figure 3) would increase sequencing throughput. We used g‐TUBEs with an Eppendorf 5,418 centrifuge to shear DNA to a target size of 20 kb by forcing it through a µm mesh. DNA shearing did not increase yield, but did affect the read‐length distribution (Table 3). Compared with nonsheared samples, the sequence read‐length distribution from sheared reads shifted to smaller values and peaked at about 11 kb (Figure 6), with an N50_Q7_ of 18 kb, compared to an N50_Q7_ of ~26 kb from the unsheared samples (Table 3). Here, a quality score of 7 (Q7) represents the default quality threshold from the basecaller. Interestingly, the median read length from the sheared DNA samples increased to 7.5 kb from 6.5 kb when compared to unsheared DNA. At the same time, low‐quality short reads were reduced in the sheared samples (Figure 6).

**Table 3:** Targeted mechanical DNA shearing does not increase sequencing throughput

Sample	Shearing	N50_Q7_ (kb)	Mean_Q7 _(kb)	Median_Q7 _(kb)	Yield (Gb)	Yield_Q7 _(Gb)
10	NO	25.8	12.4	6.2	6.0	5.9
27	NO	26	13.2	7.5	7.8	7.4
4	g‐covaris	18.4	11.8	9.5	4.8	4.7
23	g‐covaris	17.9	11.2	8.5	7.2	7.0

Note

Read‐length comparisons for unsheared and sheared samples. Comparison of N50_Q7, _mean read length_Q7_ and median read length_Q7_ of untreated samples (#10 and #27) and sheared (g‐covaris tube) samples (#4 and #23) (Figure 3).

We also tested the effect of removing DNA fragments below 20 kb by size selection using the BluePippin system in the high‐pass mode which enables the collection of DNA molecules above a certain size. When we applied the 20‐kb high‐pass filter, we were able to remove DNA fragments less than 20 kb while maintaining the high‐molecular‐weight size distribution (Figure 3). After sequencing, the read‐length N50_Q7_ increased to 35 kb from 26 kb, while the mean and median read lengths increased to 26 and 23 kb from 12 and 6.5 kb, respectively (Table 4 and Figure 3). The main drawbacks of BluePippin high‐pass size selection were the high sample loss (65%–75%), the increase in cost and prolonged sample handling.

**Table 4:** High‐pass size selection increases read‐length statistics

Sample	Size selection	N50_Q7_ (kb)	Mean_Q7 _(kb)	Median_Q7 _(kb)	Yield (Gb)	Yield_Q7 _(Gb)
10	NO	25.8	12.4	6.2	6.0	5.9
27	NO	26	13.2	7.5	7.8	7.4
2	BluePippin	35.1	26.5	23.9	3.5	3.5

Note

Read‐length comparisons for BluePippin size‐selected samples. Comparison of N50_Q7_, mean read length_Q7 _and median read length_Q7_ of untreated samples (10) and (27) and BluePippin size‐selected samples (2) (Figure 3).

### 3.3 Real time and between run evaluation

The software MinKNOW makes it possible to perform real‐time monitoring during the MinION sequencing run. Interpreting the pore signal statistics and the length graph during the first two hours of sequencing gives the user a clear idea if the run should be continued or stopped. We used this feature of MinKNOW to optimize our runs. First, we evaluated pore occupancy, defined as the ratio of “in strand” (light green) to the sum of “in strand” plus “single pores,” after one hour. A high pore occupancy (>70%) indicates successful library preparation and is predictive of a high final sequencing output. Low initial pore occupancy is predictive of low final sequencing yield. Overall we followed a traffic light system of relative pore occupancy (>70%: keep run; 30%–70%: carefully evaluate; <30%: stop run). Initially, we only stopped sequencing runs with pore occupancy below 30%. As we improved our sample handling and library preparation, we only continued runs with pore occupancy greater than 70% within 1 hr of starting the run. Otherwise, we stopped the sequencing run, washed the flowcell and loaded a new library to ensure high throughput (Supporting information Figure S1). We reasoned that low‐throughput runs were usually due to insufficient DNA molecules being ligated to sequencing adapters during the library preparation. We found that, given our DNA fragment length distribution, we had to load approximately 0.75 µg library DNA (~0.07 pmol) onto a flowcell to achieve optimal yields (Supporting information Table S1, Figure S2). To ensure sufficient adapter ligated DNA, we started library preparation with at least 4 µg of high‐quality starting DNA to account for potential losses during the SPRI bead clean‐up steps. A second pore statistic to consider is the number of unavailable pores, for example, “zero” (black), “unavailable” (light blue), or “active feedback” (pink) (Mayjonade, 2018). If these numbers increase too quickly in the first few hours of the run, it is likely that the library is contaminated and the pores are being irreversibly blocked or damaged or that the membrane has ruptured. Furthermore, the length distribution from the length graph can be easily assessed and, if unsatisfactory, the library exchanged for a separately prepared sample (Figure S1).

One key to ongoing optimization of flowcells in our laboratories was the tracking of all parameters for each sequencing run using our monitoring spreadsheet (Table S2) and a continued comparison of the output of each additional flowcell. After running each flowcell, we used ONT's Albacore 2.0 basecaller to convert the raw signal data from the MinION into DNA sequence data in fastq format. Albacore 2.0 onwards produces a sequencing_summary.txt file which contains a summary of every sequencing read, and can be used for rapid assessment of each flowcell using the minionQC script (Lanfear, Schalamun, Kainer, Wang, & Schwessinger, 2018). After basecalling each flowcell, we ran this script and examined the length and mean quality distributions of the reads in detail, and the physical performance map of the flowcell. This allowed us to continually evaluate and improve our protocols for each flowcell. Before we were halfway through our project, we were able to reliably and repeatedly obtain more than 6 Gb of data from each flowcell, with mean read lengths consistently above 12 kb.

## 4 DISCUSSION

**Figure 7:**
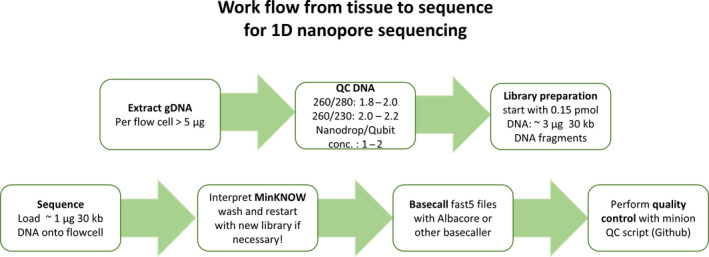
MinION nanopore sequencing workflow to optimize sequencing output. A short overview of important steps to consider when getting started, from preparation of sample to quality control of sequence output. Each box represents an essential step in this workflow. Starting with a sample optimized DNA extraction, achieving high yields of HMW DNA, followed by a quality control step using Nanodrop and Qubit values and agarose gels. Only from those samples that pass these QC requirements a sequencing library can be prepared with a minimum input amount of ~3 µg of ~30 kb DNA library for the LSK108 selecting for long‐read (ONT) library protocol. Once sufficient (~1 µg) prepared library was loaded onto the flowcell, the sequencing run can to be interpreted using the MinKNOW graphical user interphase (Supporting information Figure S1). The sequence output is basecalled either real time or after sequencing (as for this project) into fastq files. Using “sequencing_summar.txt” file from Albacore or Guppy basecaller quality control can be performed using the minion QC script (Lanfear et al., 2018) [Colour figure can be viewed at wileyonlinelibrary.com]

Here, we present a complete workflow to establish MinION long‐read sequencing (Figure 7) in any laboratory using the recalcitrant plant species eucalyptus as test case.

### 4.1 Recommendations for obtaining high‐quality high‐molecular‐weight DNA

The key starting material to every successful nanopore run is clean input DNA into the library preparation. DNA purity can be measured by Nanodrop ratios of 260/230 and 260/280 nm. Clean dsDNA displays ratios between 2 and 2.2 and 1.8 to 2.0, respectively, when all absorbance at 260 nm is caused by dsDNA. This can be assessed comparing DNA concentrations measured by dye based methods, for example, Qubit, to concentrations measured by Nanodrop. Pure dsDNA has a ratio of 1:1, and ratios of up to 1:1.5 are suitable for library preparations. Based on our observation, we recommend adhering to these DNA quality measures whenever possible, or else to assume reduced sequencing outputs. For example, in our case a reduced 260/230 nm ratio of 1.0 caused low‐sequencing yields (Table 1) because the contaminants present in the sample likely inhibit library preparation or sequencing. Hence, we also advise establishing suitable DNA extraction methods well in advance of ordering sequencing materials; our experience suggests that optimizing DNA extraction protocols can take several months. The protocols described within this manuscript, deposited on protocols.io within the MinION usergroup (https://www.protocols.io/groups/awesome-DNA-from-all-kingdoms-of-life) (Schwessinger, 2016), or published within this journal, for example, by Arsenau and colleagues provide an excellent starting point for different tissue types (Arseneau, Steeves, & Laflamme, 2017). Our general recommendation is to test different buffer conditions and precipitants, and if necessary, combine them in a sequential manner. For example, in the protocol reported in this manuscript, we first precipitate DNA with NaCl and PEG onto SPRI beads. We then clean up the DNA with a second precipitation step using ethanol with an intermediate chloroform purification step. We hypothesize that different precipitants, for example, NaCl/PEG, isopropanol, ethanol or CTAB, display varying affinities for precipitating different contaminants. By applying them in sequential manner, it may be possible to obtain clean DNA via preferential precipitation of DNA over contaminants. In addition, in our newly developed protocol, we add enzyme mixes containing pectinases and cellulases to the extraction buffer, reducing the amount of copurifying contaminants from fungal tissue (Nagar & Schwessinger, 2018b). It is important to add these enzymes during the extraction and not apply them to the final DNA suspension as most are not completely pure enzyme preparations and contain traces of DNAase activity that degrades the DNA when applied in simple solutions like TE buffer.

We (see above) and many others have reported that NaCl/PEG‐SPRI bead solutions are not always ideally suited to clean up DNA as contaminants simply coprecipitate. Following a similar logic of preferential precipitation, we hypothesize that is possible to first precipitate contaminants onto SPRI beads at low NaCl/PEG concentrations when HMW DNA stays in solution. Contaminants with higher affinity to SPRI beads and lower solubility than DNA can thereby be removed from the solution. In a subsequent step, DNA can be precipitated out of the remaining supernatant by adding more of the initial NaCl/PEG‐SPRI beads solution. This will increase the NaCl/PEG concentration and thereby precipitate the DNA out of solution onto the newly added SPRI beads (Nagar & Schwessinger, 2018b).

It is important to mention that we have had DNA preparations that fulfilled all our recommended quality control criteria but did not sequence well on the MinION. This was likely caused by “invisible” contaminants that did not absorb at the tested wavelengths (200–340 nm). However, applying a combination of the approaches suggested above enabled us to overcome this problem with our latest protocol (Nagar & Schwessinger, 2018b).

### 4.2 Achieving high‐sequencing yields with high‐quality DNA

ONT's library sequencing kits are optimized for a specific molarity of DNA molecules as they provide a fixed amount of sequencing adapters to be ligated to the free ends of the dsDNA. At the time of writing, the 1D ligation kit LSK108 requested 0.2 pmol input DNA. Because the mass of 0.2 pmol DNA depends on its fragment length, it is important to approximately estimate the mean fragment length of one's specific DNA preparation by gel electrophoresis, Tapestations or Bioanalyzer, if possible by comparison with other successful samples. DNA molecules of different length behave differently in solution, for example, diffusion rate and formation of secondary structure, which can affect the efficiency of adapter ligation and influence preferential sequencing. In general, small molecules outcompete longer DNA molecules in both cases. Hence, we stress that it is best to establish optimal DNA inputs empirically for each DNA extraction protocol, sample type and/or shearing method as shown in Figure 4. This approach can help to quickly optimize the amount of input DNA added to the ligation step.

**Figure 8:**
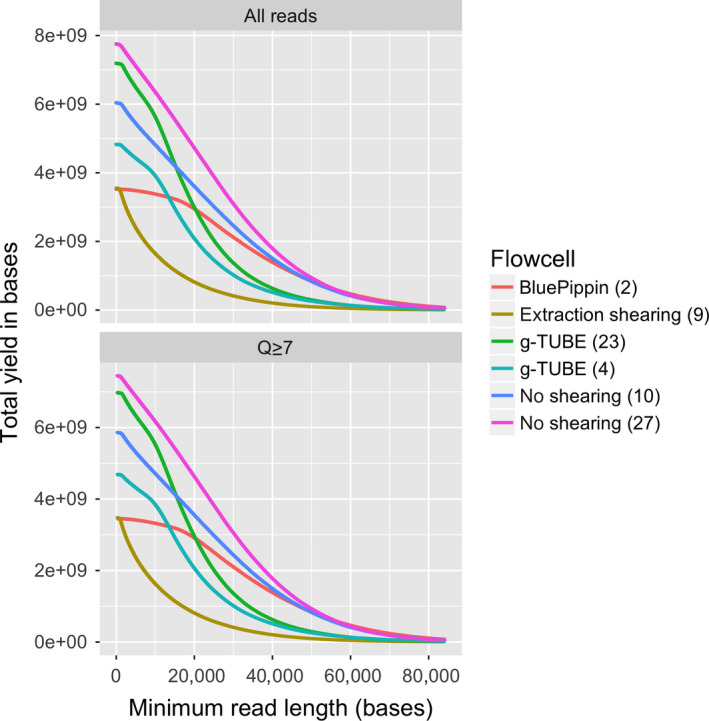
The impact of DNA extraction protocol on the yield of ONT sequencing. Each line represents a single flowcell. The *y*‐axis shows the yield of in bases, and the *x*‐axis shows the minimum read length at which the yield was calculated. For example, the yield of reads longer than 20 kb from each flowcell can be compared by comparing the height of the lines at the 20 kb point on the *x*‐axis [Colour figure can be viewed at wileyonlinelibrary.com]

Most genome sequencing projects benefit from optimizing mean, median and N50 read length. Here, we tested the impact of DNA shearing using g‐TUBEs and size selection via BluePippin on read‐length distribution and sequencing output (Figures 6 and 8). Overall, we did not employ DNA shearing or size selection in our final sequencing protocol even though they reduced the variance in read‐length distributions (Figures 6 and 8). In our case, the high‐quality sequencing results achieved with our standard protocol using the improved SPRI beads mixture did not warrant the additional time and financial investment required when incorporating g‐TUBEs DNA shearing or BluePippin size selection into our workflow. However, other projects may well benefit from maximizing read length via BluePippin size selection or of ultra‐long‐read sequencing protocols using the transposase‐based DNA library kit RAD004 (Jain et al., 2018; Quick, 2018).

One intriguing observation we made was that shearing DNA reduced the abundance of low quality “reads” (Figure 6). It is possible that removing long DNA fragments (>50 kb) reduces the incidence of long DNA molecules being stuck in the pore, at least when using the R9.5 pore chemistry. This hypothesis is supported by the observation that sheared DNA samples (4, 9, 23) have a lower tail of short low quality reads when compared to unsheared (10, 27) or BluePippin size‐selected samples (2), as shown in Figure 6 when comparing the density plots of “All reads” versus “Q ≥ 7.” This highlights that filtering reads based on their Q‐scores, as well as removing short sequencing reads, may help to avoid error propagation during downstream analyses of the data.

Lastly, user experience (*r* = 0.67) and number of available pores on the flowcell (*r* = 0.62) are the other two tracked variables (Supporting information Tables S1 and S2) that are linearly related to sequencing yields as revealed by posterior linear regression analysis (Supporting information Figure S3). Hence, experience and high‐quality flowcells in combination with high‐quality DNA will generate the best sequencing results.

### 4.3 Recommendations for genome sequencing projects

In total, the combined data from all flowcells described here (Supporting information Table S1) comprised 107 gigabases of sequence, in 12.6 million reads with an average length of 8,513 bases, a median length of 2,956 bases, and an N50 of 24,021 bases. Approximately 4 million of these “reads” were extremely short and very low quality such that the reads with an Albacore Q score ≥ 7 comprised 103 gigabases of sequence in 8.7 million reads, with an average length of 11,959 bases, a median length of 6,054 bases, and an N50 of 24,513 bases. If we assume that the genome size of *E. pauciflora *is approximately 500 Mb, approximately in line with the conserved genome sizes of other eucalypts, this represents around 200x coverage of the genome (Grattapaglia et al., 2012). The length distribution of the reads is such that we generated 61 gigabases of reads (or ~122x coverage) longer than 20 kb, and 15 Gb of reads (or ~30x coverage) longer than 50 kb. These read distributions are expected to be more than sufficient to assemble a high‐quality reference genome, particularly if they are combined with high‐accuracy short read data to polish minor errors (Jiao & Schneeberger, 2017; Michael et al., 2018; Schmidt et al., 2017).

## 5 CONCLUSION

The field of nanopore sequencing is extremely fast moving. Updates on sequencing library kits and the MinKNOW software made some of the specific recommendations of the initial version of this manuscript less applicable. At the same time, much of the general pointers and advice will be useful to new laboratories starting out with MinION sequencing independently of updates in sequencing chemistry and software. Overall we highlight the importance of clean high‐molecular‐weight DNA for successful sequencing runs and provide detailed wet laboratory DNA extraction and purification protocols that include size selection. Once established under regular laboratory conditions, some of these protocols may also be adaptable to sequencing in the field by reducing their complexity. All of these protocols, and many others applicable to different starting material provided by other community members, are freely available on the open‐access protocol sharing repository protocols.io in form of a MinION user group (https://www.protocols.io/groups/awesome-DNA-from-all-kingdoms-of-life) (Schwessinger, 2016). We encourage others to contribute to this open science platform to accelerate research and for the community to save costs when establishing long‐read DNA sequencing in their own laboratories. High‐quality “living” protocols with careful run and run‐to‐run evaluations as described here (see Supporting information Table S2 and R script on https://github.com/gringer/minion-user-group for inspiration) will facilitate knowledge generation instead of constant “reinvention of the wheel” (Lanfear et al., 2018).

## 6 DATA ACCESSIBILITY STATEMENT

All data in this manuscript are available online. The raw fastq files of all sequencing runs are deposited in the Short Read Archive with SRA project ID SRP14560 and BioProject ID PRJNA450887. The individual runs can be found with run IDs SRR7153074, SRR7153075, SRR7153076, SRR7153077, SRR7153078, SRR7153079, SRR7153080, SRR7153081, SRR7153082, SRR7153083, SRR7153094, SRR7153095, SRR7153096, SRR7153097, SRR7153098, SRR7153099, SRR7153100, SRR7153101, SRR7153102, SRR7153103, SRR7153110, SRR7153112, SRR7153113, SRR7153114 and SRR7153115. See Supporting information Table S1 for details of matching “Original Sample name” with specific SRA entries. The code to analyse the Supporting information Table S2 can be found at https://github.com/gringer/minion-user-group .

The three main protocols (1‐3) of this manuscript can be accessed under the following digital object identifiers; dx.doi.org/10.17504/protocols.io.khkct4w, dx.doi.org/10.17504/protocols.io.idmca46, and dx.doi.org/10.17504/protocols.io.n7hdhj6.
