# Supplementary material for: Harnessing the MinION: An example of how to establish long‐read sequencing in a laboratory using challenging plant tissue from Eucalyptus pauciflora
Source: Mol Ecol Resour. 2018 Oct 5;19(1):77–89. doi: 10.1111/1755-0998.12938 (PMC7380007; doi:10.1111/1755-0998.12938)
Supplement: Supplementary file 3 [file MEN-19-77-s003.pdf]

MOLECULAR ECOLOGY RESOURCES

Supplemental Information for:

Harnessing the MinION: An example of how to establish long-read sequencing in a laboratory using challenging plant tissue from Eucalyptus pauciflora

Authors:
Miriam Schalamun[a,b], Ramawatar Nagar[a], David Kainer[a], Eleanor Beavan[a], David Eccles[c], John P. Rathjen[a], Robert Lanfear[a,$], Benjamin Schwessinger[a,$]

Research School of Biology, The Australian National University, Acton 2601, ACT, Australia[a]; Current address: University of Natural Resources and Life Sciences, 1190 Vienna, Austria[b], Malaghan Institute of Medical Research, Wellington, New Zealand[c]
[$] Corresponding authors:
Robert Lanfear, email: rob.lanfear@anu.edu.au, Benjamin Schwessinger, email: benjamin.schwessinger@anu.edu.au

Table of Contents:

**Supplemental Figure 1. Real time analysis of sequencing runs via the MinKNOW graphical user interface**	**Page 2**
**Supplemental Figure 2. Effect of loaded library amount on normalized sequencing yield per pore**	**Page 3**
**Supplemental Figure 3. Sequencing yield depends on quality control statistics**	**Page 4**
**Protocol 1. High molecular weight gDNA after Mayjonade et al. optimised for eucal nanopore sequencing**	**Page 5-15**
**Protocol 2: DNA size selection (>1kb) and clean up using an optimized SPRI beads mixture**	**Page 16-22**
**Protocol 3: DNA size selection (>3-4kb) and purification of DNA using an improved homemade SPRI beads solution**	**Page 23-29**

MOLECULAR ECOLOGY
RESOURCES

A) B)

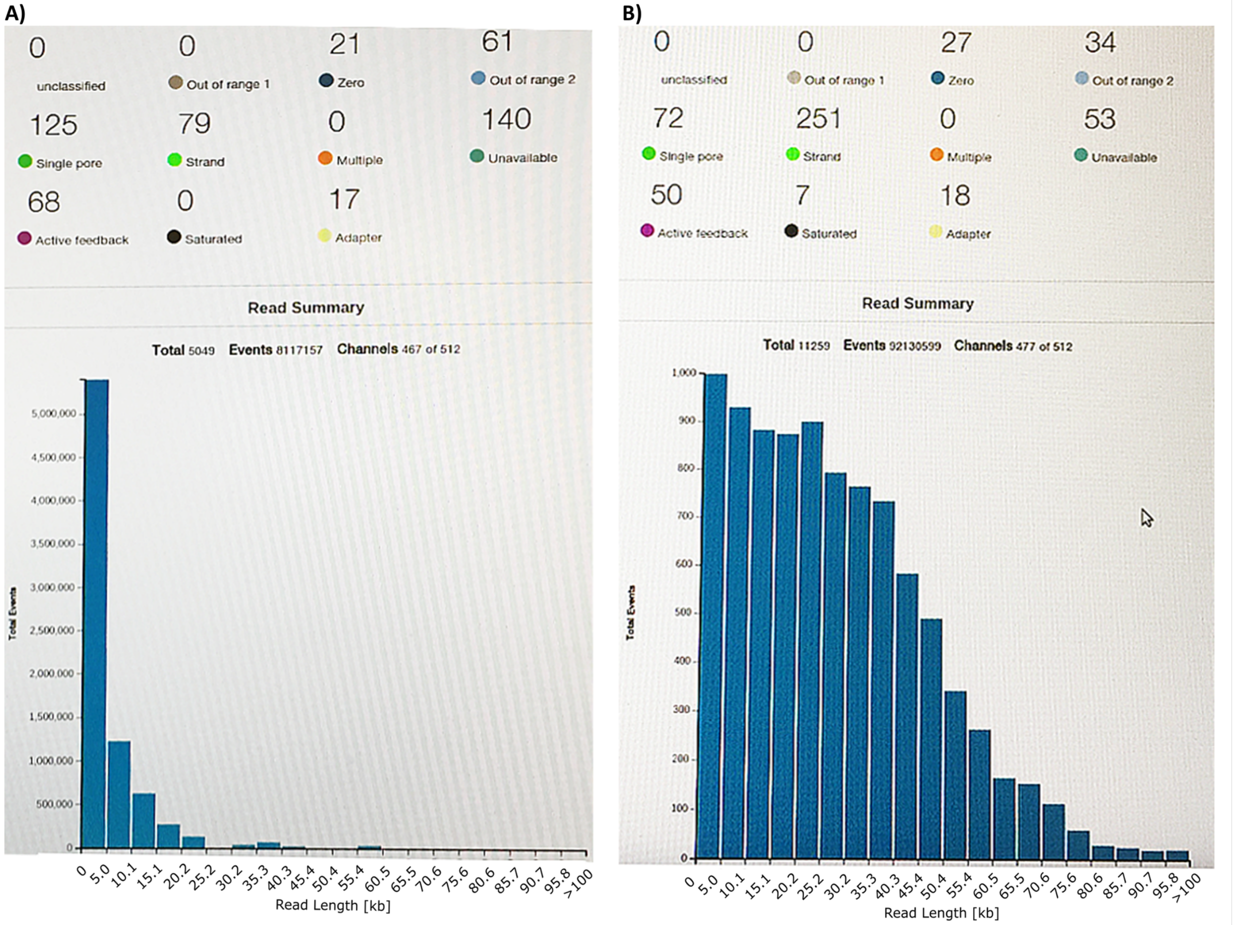

Supplemental Figure 1. Real time analysis of sequencing runs via the MinKNOW graphical user interface

Both panels (A and B) show the MinKNOW interface two hours into a run. Panel A illustrates an unsatisfactory sequencing run where read length is short, pore occupancy poor (~40%) and many pores are not available for sequencing any more (see main text for details). This run was aborted after two hours to not to waste this flow cell and to reload an improved library. Panel B illustrates a satisfactory sequencing run with excellent read length distribution, good pore occupancy (~80%), and most pores still readily available for sequencing. Whereas read length distribution is displayed in events (here kilo events for a better readability).

MOLECULAR ECOLOGY
RESOURCES

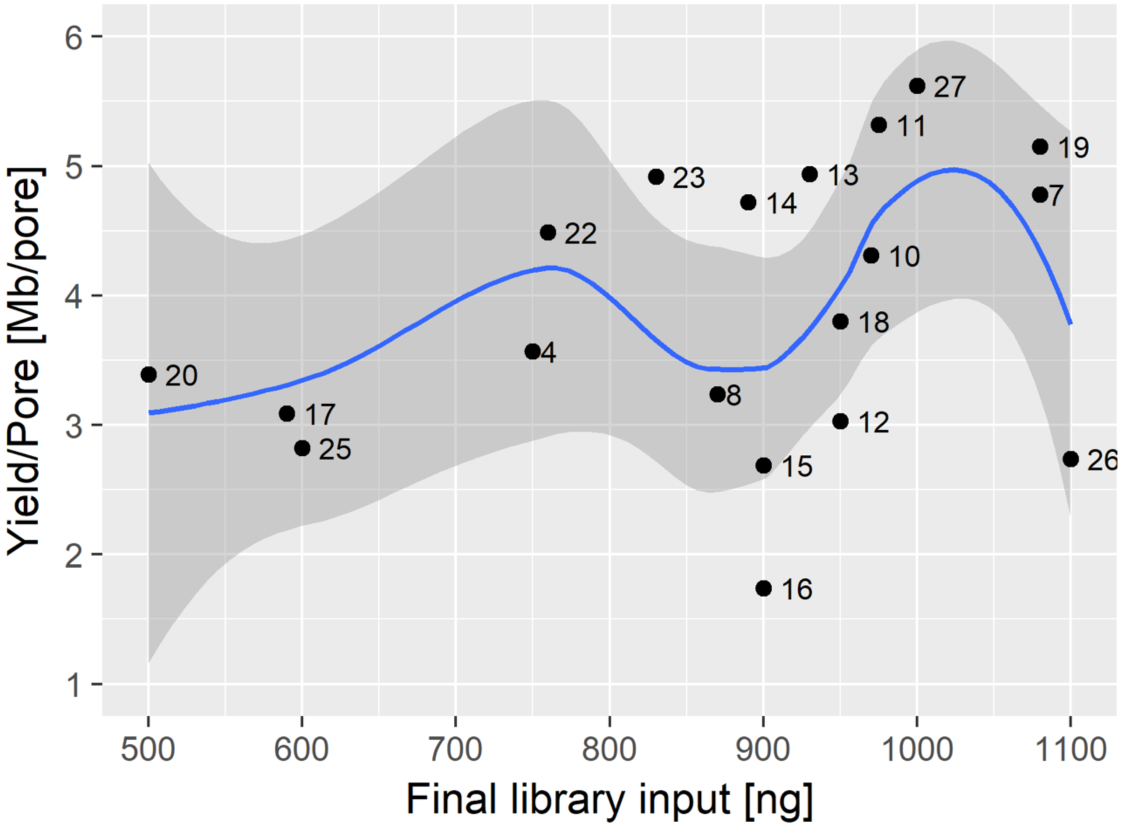

Supplemental Figure 2. Effect of loaded library amount on normalized sequencing yield per pore

DNA library loaded [µg] on the flowcell (x-axis) versus sequencing yield normalized by available pores during flowcell QC [Mb/pore].

MOLECULAR ECOLOGY
RESOURCES

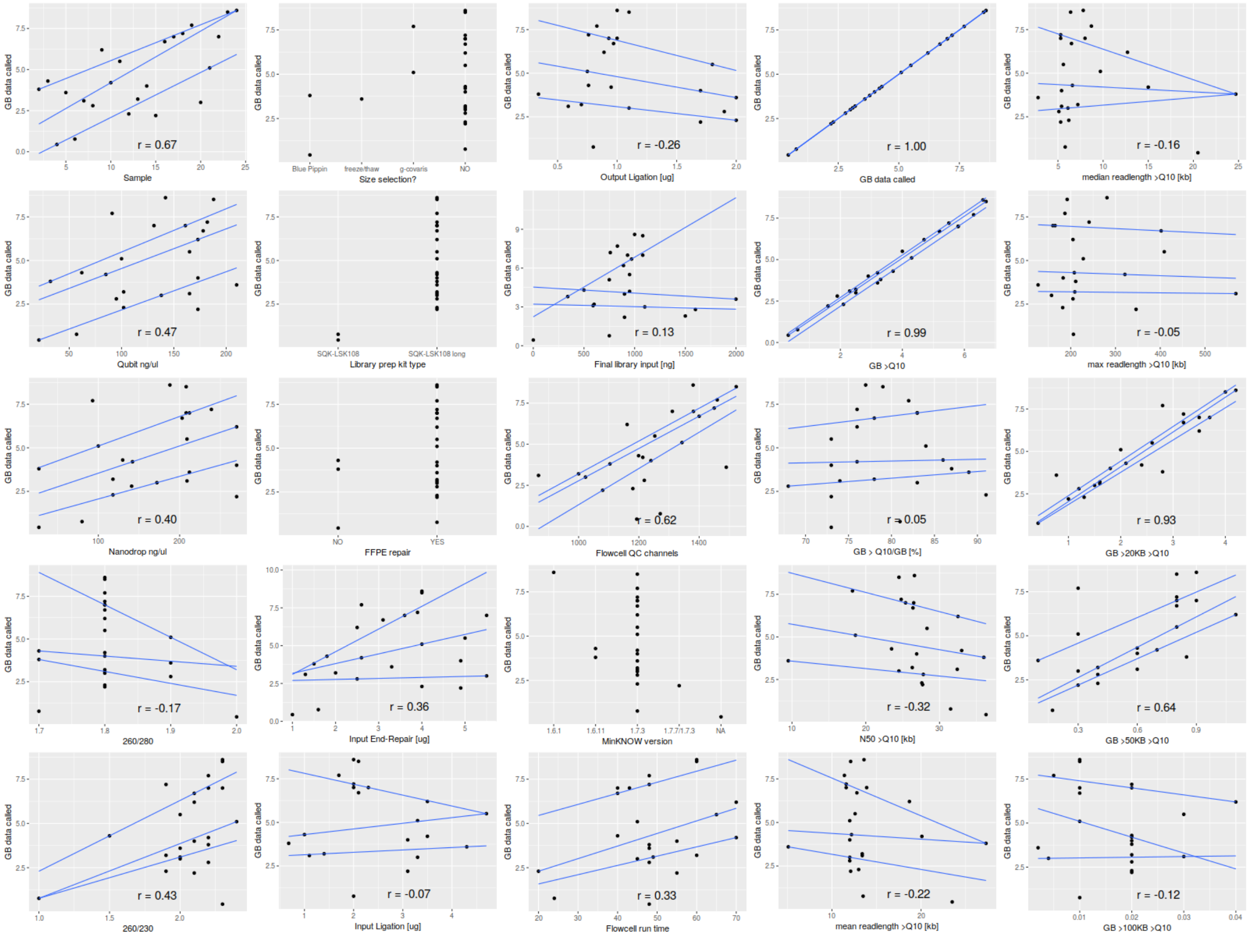

Supplemental Figure 3. Sequencing yield depends on quality control statistics
Comparison of sequencing yield (as GB of called data) versus all measured QC statistics. Positive correlations that are not directly related to yield are seen with sample number, the number of channels seen during flow cell QC, and the measured initial input library concentration. Lines indicate fitted quantile regression lines at 25%, 50% and 75% (i.e. lower quartile, median, and upper quartile respectively). The script for plotting the quality control statistics vs sequencing yield is provided on github (https://github.com/gringer/minion-user-group).

High molecular weight gDNA extraction after Mayjonade et al. optimised for eucalyptus for nanopore sequencing Version 9

Miriam Schalamun,Benjamin Schwessinger

Abstract

Extraction of high quality DNA for long read sequencing e.g. the Oxford Nanopore
Optimized for DNA extraction from eucalyptus grandis and eucalyptus pauciflora.

This protocol contains an optional Chloroform clean up step which is necessary for eucalyptus but might not be for other tissue.

For long DNA fragments don't vortex the DNA sample.

Citation: Miriam Schalamun,Benjamin Schwessinger High molecular weight gDNA extraction after Mayjonade et al. optimised for eucalyptus for nanopore sequencing. **protocols.io**
dx.doi.org/10.17504/protocols.io.khkct4w
Published: 30 Oct 2017

Guidelines

Modified from the protocol of Baptiste Mayjonade, Jérome Gouzy, Cécile Donnadieu, Nicolas Pouilly, William Marande, Caroline Callot, Nicolas Langlade and Stéphane Munos, High molecular weight gDNA extraction, Bio Techniques, Vol. 61, No. 4, October 2016, pp. 203-205.

Link to webpage:
http://www.biotechniques.com/BiotechniquesJournal/2016/October/Extraction-of-high-molecular-weight-genomic-DNA-for-long-read-sequencing-of----single-molecules/biotechniques-365135.html

When citing please make sure to also mention the original Mayjonade et al. protocol as described above.

Thank you Benjamin Schwessinger for the constant support in the lab and outside!

Reagents required
(stock solutions)

Lysis buffer:
10% PVP 40
10% PVP 10
5M NaCl
1M TRIS pH 8
0.5 M EDTA
20% SDS
Sodium metabisulfite (190.1 g/mol)

This is an open access protocol distributed under the terms of the Creative Commons Attribution License, which permits unrestricted use, distribution, and reproduction in any medium, provided the original author and source are credited

Molecular biology grade water

Beads solution (adapted from Philippe Jolivet and Joseph W. Foley, 2015 - Solutions for purifying nucleic acides by solid-phase reversible immobilization (SPRI)) very useful tips about preparing the high concentrated PEG solutions can be found - definitely worth a look!

Sera-Mag SpeedBeads (GE Healthcare, 65152105050250)
50% PEG 8000
5 M NaCl
1 M Tris-HCl pH 8
0.5 M EDTA pH 8
molecular biology grade water

Other solutions:
5M Potassium Acetate pH 7.5
Ethanol 70%
TE-Buffer (10 mM Tris, 1 mM EDTA)

Additional for Clean up step:
Chloroform:Isoamylalcohol (24:1)
Natriumacetate 3 M
Ethanol 100 %
Ethanol 70 %
TE-Buffer

Enzymes
RNAse A (1000 U/ml, Thermo Fisher EN0541)
Proteinase K (800U/ml, NEB P81072)

Materials:
TissueLyser II (Qiagen)
Thermomixer
Magnetic rack for Eppendorf tubes

Before start

Prepare beads stock solution

This is the same beads stock solution as in the size selection protocol:
https://www.protocols.io/view/dna-size-selection-1kb-and-clean-up-using-an-optim-ir3cd8n

For 10 mL:

This is an open access protocol distributed under the terms of the Creative Commons Attribution License, which permits unrestricted use, distribution, and reproduction in any medium, provided the original author and source are credited

Final	stock	Input
10 mM Tris-HCl	1 M	100 µl
1 mM EDTA pH 8	0.5 M	20 µl
1.6 M NaCl	5 M	3.2 ml
11% PEG 8000	50% (w/v)	2.2 ml
0.4% beads (v/v)	100%	40 µl
Milliq Water		4.44 ml

**Because there were some questions considering the beads concentration:*

- 0.4% beads final are 40 µL of the Sera Mag speed beads in a total Volume of 10 mL 'beads stock solution'- thats not much but you definitely should see them in the solution!

- there is no issue in increasing the beads concentration to e.g. 1% (100 µL in the final 10 mL), there should be no change in size selection, the beads have a binding capacity of 5 µg/ml anyway. I tested the different beads concentrations and there was no difference between final 0.4% - 2% beads, I just took the 0.4% because I needed a lot of beads solution in total.

-Make sure to use the Sera-Mag SpeedBeads and not the AMPure XP beads for preparing the beads stock solution. The AMPure XP beads are used straight (at 0.45V) without further manipulation.

1. Frist combine only Water, Tris-HCl, EDTA and NaCl in a 50 mL tube.

2. Vortex Sera-Mag SpeedBeads (GE Healthcare, 65152105050250) very well and pipette 40 µl into a 1.5 ml tube, put it on the magnetic rack and wait until solution has cleared up and all beads have bound to the back of the tube

3. Wash beads by removing supernatant and adding 1.5 ml milliq water

4. Take tube of the magnet, mix well, spin down in a microcentrifuge and put back on the magnet

5. Wait for beads to assemble at the back of the tube

6. Pipette off and discard supernatant

7. Repeat washing (steps 3 - 6) 3 more times

This is an open access protocol distributed under the terms of the Creative Commons Attribution License, which permits unrestricted use, distribution, and reproduction in any medium, provided the original author and source are credited

8. After pipetting of the supernatant the last time take off tube from the magnet and add 40 µl of the previous (step 1) prepared stock solution, mix well, spin down and pipette everyting into the remaining stock solution in the 50 mL tube and mix

9. Now the 2.2 ml 50% PEG can be added to the stock solution, which after vortexing very well is ready for use.
 Be careful to actually pipette 2.2 ml as solution is very viscous, but the final concentration of PEG is crucial for the clean up to work properly.

Always use fresh lysis buffer and fresh 70 % Ethanol

Clean up steps 26 - 41 are optional and are only necessary in recalcitrant tissue like eucalyptus.
The extraction and clean up step can also be done on seperate days if DNA is stored at 4°C.

Protocol

Prepare lysis buffer

Step 1.
Always prepare fresh lysis buffer before extraction for optimal results (especially the DTT should be added fresh)
For 10 mL Lysis Buffer:

Final	stock	Input
1% PVP 40	10%	1 mL
1% PVP 10	10%	1 mL
500 mM NaCl	5 M	1 mL
100 mM TRIS pH 8	1 M	1 mL
50 mM EDTA	0.5 M	1 mL
1.25% SDS	20%	625 µL
1% (w/v)! Sodium metabisulfite	190.1 g/mol	0.1 g
5 mM Dithiothreitol (DTT)	1 M	50 µL
Milliq Water		4.3 mL

Heat lysis buffer to 64 °C for 30 minutes. (This is important for the DNase heat inactivation)

After cooling down to room temperature add per 1 mL lysis buffer 1 uL RNase A (in this case 10 µL)

ANNOTATIONS

This is an open access protocol distributed under the terms of the Creative Commons Attribution License, which permits unrestricted use, distribution, and reproduction in any medium, provided the original author and source are credited

Hi,

Is there any information if I can use CTAB instead of SDS? Will it have any problem with beads?

Prepare tissue

Step 2.

In the meantime prepare 2 mL Eppendorf tubes with 1-2 metal beads (5 mm) and 100 mg of tissue and transfer tubes into liquid nitrogen.
This is the easiest with fresh tissue because freeze thawing is avoided during cutting. But if there is no other way, tissue frozen in liquid nitrogen and making sure tissue stays frozen also works.

AMOUNT

100 mg Additional info:
Tissue

Grinding

Step 3.

Before ginding make sure tissue is completely frozen in liquid nitrogen and do the grinding steps as quickly as possible to avoid freeze thawing. The grinding reck can also be frozen to ensure that.

Grind tissue using an automated grinder (Qiagen TissueLyzer II) for 40 seconds (actual grinding time may differ from tissue to tissue)

Extraction I

Step 4.

Add 700 uL of preheated buffer and mix by inverting tube until no frozen clumps of tissue are left (This can take up to a few minutes, but it's worth it)

AMOUNT

700 µl Additional info:
Preheated buffer

Extraction I

Step 5.

Incubate at 37°C in a thermomixer shaking at 400 rpm (slowly) for 20 minutes

Extraction I

Step 6.

Add 10 uL Proteinase K per tube and incubate for another 20 - 30 min at 37°C in the thermomixer

AMOUNT

10 µl Additional info:
Proteinase K

Extraction I

This is an open access protocol distributed under the terms of the Creative Commons Attribution License, which permits unrestricted use, distribution, and reproduction in any medium, provided the original author and source are credited

Step 7.

Take the tubes out of the termomixer and cool down on ice for 5 minutes

Extraction I

Step 8.

Add 210 uL (0.3 volumes) of 5M Potassium Acetate and mix by inverting the tube 20 times and then immediately keep on ice at 4°C

AMOUNT

210 µl Additional info: 5M Potassium Acetate

ANNOTATIONS

Is there a reason why Potassium Acetate is used here instead of Sodium Acetate (as suggested in the Clean-Up stage)?

Extraction I

Step 9.

Centrifuge at 8000g for 12 minutes at 4°C

Extraction I

Step 10.

Transfer the supernatant (600 uL) to a new 1.5 mL tube without disturbing the pellet

Extraction I

Step 11.

Add 1 volume (600 uL) of beads solution previously prepared (make sure beads are at room temperature and well homogenized via vortexing for approximatelly 30 seconds)

AMOUNT

600 µl Additional info: Beads solution

NOTES

Only vortex the beads alone before adding to the sample
Do NOT vortex the sample containing the DNA

ANNOTATIONS

Could you give more details about the contents of the bead solution?

Extraction I

This is an open access protocol distributed under the terms of the Creative Commons Attribution License, which permits unrestricted use, distribution, and reproduction in any medium, provided the original author and source are credited

Step 12.

Mix by inversion and then incubate on a rotor for 10 minutes at RT

(In the meantime put TE buffer into waterbath at 50°C so that its preheated later)

Extraction I

Step 13.

Spin down the tube for 1 second

Extraction I

Step 14.

Place the tube in a magnetic rack for 5 minutes (until beads are stuck to the wall of the tube and solution becomes clear)

Extraction I

Step 15.

Remove the supernatant without disturbing the beads

Extraction I

Step 16.

Add 1 mL of fresh 70 % Ethanol and wait for 30 seconds

AMOUNT

1 ml Additional info: 70 %
Ethanol

Extraction I

Step 17.

Remove supernatant without disturbing the beads

Extraction I

Step 18.

Repeat the washing steps 15 - 16 once more

GOTO

Repeat washing -> go to step #15

Extraction I

Step 19.

Spin down the tube for 1 second and place the tube on the magnetic rack to remove the ramaining Ethanol

Extraction I

Step 20.

This is an open access protocol distributed under the terms of the Creative Commons Attribution License, which permits unrestricted use, distribution, and reproduction in any medium, provided the original author and source are credited

Let the beads air-dry for 30 seconds, but not longer beaucse this would decrease elution efficiency

Extraction I

Step 21.

Add 50 uL of TE buffer preheted to 50°C and resuspend the beads by flicking the tube (make sure they are not aggregated anymore)

AMOUNT

50 µl Additional info: TE buffer

NOTES

This volume can be changed to the amount needed (between 20 - 100 µl are most usefull to handle)

Extraction I

Step 22.

Incubate the resuspended beads for 10 minutes at room temperature

(to make sure the DNA can go back into elution)

Extraction I

Step 23.

Spin down the tube for 1 second and place the tube in the magnetic rack and incubate for 5 -10 minutes (until solution becomes clear)

Extraction I

Step 24.

Transfer the supernatant (eluted DNA) into new tube

QC I

Step 25.

Meassure DNA concentration with a Qubit and absorbance with a NanoDrop.
Aiming for:
Qubit/Nanodrop: 0.5 - 1.0
260:280: 1.8 - 2.0
260:230: 2 - 2.2

For tissue with less secondary metabolites and oils, the purity of the DNA sample most likely should be good and similar to the values described above.

For recalcitrant tissue though, like Eucalyptus the following clean up step is recommended.

Clean up

This is an open access protocol distributed under the terms of the Creative Commons Attribution License, which permits unrestricted use, distribution, and reproduction in any medium, provided the original author and source are credited

Step 26.

Transfer the DNA solutions into one tube and add TE-Buffer to make up a total volume of 500 µL

If the extraction is done with multiple tubes at a time (which usually is the case), then after the elution step all the eluted DNA can just be pipettet into one tube.

Clean up

Step 27.

Add 500 µL (1 V) Chloroform:Isoamylalcohol (24:1) and invert tube about 100 times (2 minutes at least)

AMOUNT

500 µl Additional info: Chloroform:Isoamylalcohol (24:1)

Clean up

Step 28.

Centrifuge tube to separate the phases at 8000g at 4°C for 10 minutes

Clean up

Step 29.

Transfer the upper phase (DNA) into a new tube and discard the lower chloroform phase

(depending on how good the transfer worked out one round is usually enough but if some of the intermediate phase has been transferred, steps 28 -29 can be repeated)

Clean up

Step 30.

Add 50 µL (0.1 V) 3 M Sodium Acetate (NaAc) and mix by inverting tube 10-20 times

AMOUNT

50 µl Additional info: 3 M Sodium Acetate

ANNOTATIONS

reps/time? as above with 100 inversions? just a few? thanks

Clean up

Step 31.

Add 500 µL (1 V) of 100% Ethanol and mix by inverting tube carefully a few times and then let incubate at 4°C for 5 - 10 minutes.

Depending on DNA concentration (for me at 100ng/µL) DNA starts to precipitate but if the sample is very

This is an open access protocol distributed under the terms of the Creative Commons Attribution License, which permits unrestricted use, distribution, and reproduction in any medium, provided the original author and source are credited

clean, which is desirable, this will be seethrough. So only is recognisable by a thickening of the solution and probably air bubbles bound to precipitate (at least that what it looks like to me).

AMOUNT

500 µl Additional info:
100% Ethanol

Clean up

Step 32.

Centrifuge at 4°C and 5000g for 2 minutes.

(If nothing precipitates out of solution after that, centrifuge for 10 minutes at 10000g)
But the short centrifugation time and lower speed are used to select for longer fragments in the beginning.

Clean up

Step 33.

Pipette supernatant off and make sure to do it on the opposite side than the pellet is supposed to be.

If the the pellet was seethrough I would recommend pipetting the supernatant into a new tube just to make sure that if nothing precipitated out and no pellet is seen the supernatant can be centrifuged again at a higher speed and longer.

Sometimes the "pellet" for me is just a seethrough smear along the tube (due to the low speed I guess)

Clean up

Step 34.

Add 1 mL 70 % Ethanol to pellet to wash off salt

AMOUNT

1 ml Additional info: 70%
Ethanol

Clean up

Step 35.

Centrifuge at 4°C and 8000 g for 5 minutes (here the higher speed shouldn't matter anymore because DNA is pelleted already)

Clean up

Step 36.

Remove supernatant

This is an open access protocol distributed under the terms of the Creative Commons Attribution License, which permits unrestricted use, distribution, and reproduction in any medium, provided the original author and source are credited

Clean up

Step 37.

Repeat steps 35 - 37 once more and make sure all Ethanol is pipetted off

GOTO

Repeat steps 35-37 -> go to step #35

Clean up

Step 38.

Let air dry (for last removal of Ethanol) for 2 - 5 minutes
(here the drying time is not as critical as in the beads drying step as the DNA pellet should easier dissolve than the beads)

Clean up

Step 39.

Add 50 µL of preheated (to 50°C) 10 mM Tris (pH 8) or TE-Buffer and elute DNA as long as necessary for the whole pellet to dissolve.
If it's a large pellet I let it dissolve overnight at room temperature

AMOUNT

50 µl Additional info: 10 mM Tris (pH 8) or TE-Buffer

QC II

Step 40.

Measure Qubit and Nanodrop values again (as in step 25)

GOTO

Qubit and Nanodrop values -> go to step #25

This is an open access protocol distributed under the terms of the Creative Commons Attribution License, which permits unrestricted use, distribution, and reproduction in any medium, provided the original author and source are credited

DNA size selection (>1kb) and clean up using an optimized SPRI beads mixture

Miriam Schalamun,Benjamin Schwessinger

Abstract

This protocol describes a clean up and size selection method for nucleic acids (tested on DNA) to deplete and remove fragments below 1 - 2 kb.
The success of this depends on the cleanliness of your sample (it doesn't have to be super clean but a whole lot of contaminants make working with the beads more difficult, diluting the sample out before usage can help with that).

The concentrations of PEG and NaCl and the volume of the beads solution are crutial for recovery and proper removal of small fragments.
As a basic guidline it can be said: more PEG and NaCl - higher recovery but hence less removal of small fragments and the other way round. I found for my samples (eucalyptus) that with 1 volume of the beads solution respectively to DNA sample I'm on the safe side recovery wise, but if I want to make sure to get rid of more smaller fragments I use 0.8 volumes.

So in numbers that means:
Final reaction concentration of PEG8000:
1 V: 5.5%
0.8 V: 4.8%

Final reaction concentration of NaCl:
1V: 0.8 M
0.8V: 0.7 M

Citation: Miriam Schalamun,Benjamin Schwessinger DNA size selection (>1kb) and clean up using an optimized SPRI beads mixture. **protocols.io**
dx.doi.org/10.17504/protocols.io.idmca46
Published: 01 Jul 2017

Guidelines

Beads solution adapted from Philippe Jolivet and Joseph W. Foley, 2015 - Solutions for purifying nucleic acides by solid-phase reversible immobilization (SPRI))
Link: http://www.openwetware.org/images/f/f8/SPRI_buffers_v2_2.pdf
There really accurate and helpfull recipies for making up every ingredient of the beads solution can be found, I just adapted the concentrations for my needs.

Thank you Benjamin Schwessinger for supporting and encouraging me to try every single concentration combination out there to find a simple and well working one.

This is an open access protocol distributed under the terms of the Creative Commons Attribution License, which permits unrestricted use, distribution, and reproduction in any medium, provided the original author and source are credited

I also use the same beads solution for the MinION sequencing library preparation.

Before start

Before doing this clean up on your whole (maybe very precious) sample I would recommend testing it out on a smaller amount (300 - 500ng) to make sure the beads work for you.

- For making the beads solution stock take Sera-Mag SpeedBeads (GE Healthcare, PN 24152105050250) out of the fridge and let warm up to room temperature

- For further clean ups always take the beads stock solution out of the fridge at least 15 minutes before usage to let it warm up to room temperature and mix (vortex) very well until soluion looks homogeneous and no bead clumps are visible anymore (that is very important)

- Always make fresh 70 % Ethanol

- Preheat your elution buffer of choice (TE-Buffer, Tris 10 mM, Water..) to 50° until usage

Protocol

Make beads stock solution

Step 1.

For 10 mL beads stock solution:

Final	stock	Input
10 mM Tris-HCl	1 M	100 µl
1 mM EDTA pH 8	0.5 M	20 µl
1.6 M NaCl	5 M	3.2 ml
11% PEG 8000	50% (w/v)	2.2 ml
0.4% beads (v/v)	100%	40 µl
Milliq Water		4.44 ml

Frist combine only Water, Tris-HCl, EDTA and NaCl in a 50 mL tube.

Make beads stock solution

Step 2.

Vortex Ser-Mag SpeedBeads (GE Healthcare, PN 24152105050250) very well and pipette 40 µl into a 1.5 ml tube, put it on the magnetic rack and wait until solution has cleared up and all beads have bound to the back of the tube

ANNOTATIONS

This is an open access protocol distributed under the terms of the Creative Commons Attribution License, which permits unrestricted use, distribution, and reproduction in any medium, provided the original author and source are credited

Apologies we got the product number wrong. This should be SpeedBeads magnetic carboxylate moditifed particles GE Healthcare, 65152105050250.

Make beads stock solution

Step 3.

Wash beads by removing supernatant and adding 1.5 ml milliq water

Make beads stock solution

Step 4.

Take tube of the magnet, mix well, spin down in a microcentrifuge and put back on the magnet

Make beads stock solution

Step 5.

Wait for beads to assemble at the back of the tube

Make beads stock solution

Step 6.

Pipette off and discard supernatant

Make beads stock solution

Step 7.

Repeat washing (steps 3 - 6) 3 more times

Make beads stock solution

Step 8.

After pipetting of the supernatant the last time take off tube from the magnet and add 40 µl of the previous (step 1) prepared stock solution, mix well, spin down and pipette everyting into the remaining stock solution in the 50 mL tube and mix

Make beads stock solution

Step 9.

Now the 2.2 ml 50% PEG can be added to the stock solution, which after vortexing very well is ready for use.

Be careful to actually pipette 2.2 ml as solution is very viscous, but the final concentration of PEG is crucial for the clean up to work properly.

Clean up

Step 10.

Bring you DNA sample in a 1.5 ml tube to comfortable pipetable volume (I usually do it with some volume

This is an open access protocol distributed under the terms of the Creative Commons Attribution License, which permits unrestricted use, distribution, and reproduction in any medium, provided the original author and source are credited

between 20 - 200 µl) and if you know already that your sample contains a lot of contaminants and/or DNA (hence a really viscous solution) diluting it out and splitting into two tubes can make life easier.

(A lot of contaminants can also clump around the beads which makes binding to the magnet slower and sometimes pipetting off the supernatant really difficult)

Clean up

Step 11.

Make sure to know the exact volume of your sample and add 1 V of that (or 0.8 V for removal of more smaller fragments but also higher risk of less recovery) of well homogenised, room temperature beads solution and mix by flicking the tube

Clean up

Step 12.

Place tube on a rotor (or mixer if you don't have one) and mix for 10 minutes

Clean up

Step 13.

Spin down tube in the microcentrifuge and place on the magnet

Clean up

Step 14.

Wait until beads have moved to the back of the tube and the solution becomes clear

(depending on viscosity of the solution that can take between 1 min to much longer like 1h, if after that the beads look like they are stuck in a big cloud and just don't properly bind to the magnet I add same amount of buffer and beads (the ratio always has to stay the same!) mix again and the put it back on the magnet - usually that solved the problem. But you probably will never have that problem, I just worked with really contaminated samples (plants))

Clean up

Step 15.

Remove and discard supernatant

Clean up

Step 16.

Wash beads with fresh 70% Ethanol by adding 1 - 1.5 mL to the opposite side than where the beads bind

This is an open access protocol distributed under the terms of the Creative Commons Attribution License, which permits unrestricted use, distribution, and reproduction in any medium, provided the original author and source are credited

and wait 30 seconds

Clean up

Step 17.

Remove and discard Ethanol

Clean up

Step 18.

Repeat washing once more (steps 15 - 18)

(Don't remove tube from magnet during the washing steps until here)

Clean up

Step 19.

For the last removal of Ethanol make sure that all the Ethanol is removed, therefore take tube off the magnet , spin down for a second and place back onto the magnet, like that also the last drops of Ethanol can be pipetted off

Clean up

Step 20.

Let beads air dry for a maximum of 30 seconds or else elution will be difficult

Clean up

Step 21.

Add 50 ul (or in whatever final volume and concentration the sample is needed) of preheated to 50°C 10 mM Tris (or TE-Buffer)

Clean up

Step 22.

Make sure the beads are resuspend properly by flicking the tube gently and spinning it down - the solution will be homogeneous and brown

(I put it in a 50°C Thermoblock for about 20 seconds to encourage the elution reaction)

Clean up

Step 23.

Spin down the tube before placing it on the magnet again and wait until the beads have bound the the back of the tube (that can again take its time especially if the sample contains really long DNA fragments) Depending on the sample that will take between 1 minute to a few hours, I usually wait 5 - 10 minutes

Clean up

This is an open access protocol distributed under the terms of the Creative Commons Attribution License, which permits unrestricted use, distribution, and reproduction in any medium, provided the original author and source are credited

Step 24.

When the solution has cleared up completely, pipette the supernatant to a fresh tube and discard beads.

Quality control

Step 25.

Meassure the DNA concentration with a Qubit.

The recoveries for HMW DNA > 20kb should lie between 60 - 90%

Quality control

Step 26.

Run a 0.8% agarose gel

80 ng DNA per sample, normalised to 10 ul per lane

run for 45 min in 1 x TBE buffer

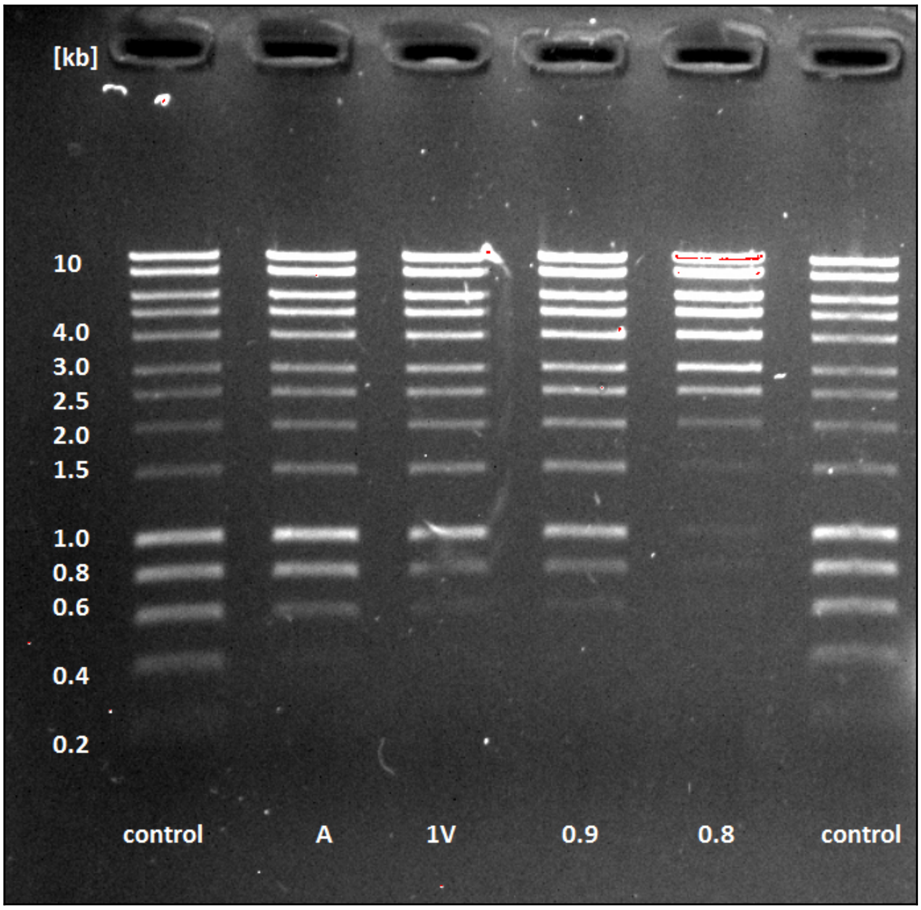

Lanes:

control: untreated 10kb hyperladder

A: 0.45 AMPure Beads XP beads (Beckman Coulter). This is the standard dilution used in PacBio and Oxford Nanopore protocols.

1: 1 volume of beads solution from this protocol

This is an open access protocol distributed under the terms of the Creative Commons Attribution License, which permits unrestricted use, distribution, and reproduction in any medium, provided the original author and source are credited

0.9: 0.9 volumes
0.8: 0.8 volumes

This is an open access protocol distributed under the terms of the Creative Commons Attribution License, which permits unrestricted use, distribution, and reproduction in any medium, provided the original author and source are credited

DNA size selection (>3-4kb) and purification of DNA using an improved homemade SPRI beads solution.

Ramawatar,Benjamin Schwessinger

Abstract

Most noncommercial tradition DNA extraction protocols result in a crude DNA preparation. If the DNA is intended to be used for a high-end application like Nanopore sequencing, it requires a thorough clean-up and size selection before it could be used for sequencing.
Solid Phase Reversible Immobilisation (SPRI) magnetic beads is a quick and convenient way of purifying and size selecting intact double-stranded DNA from crude DNA. Most commercially available SPRI beads based DNA purification mix is quite expensive so our lab endeavored to develop an inexpensive beads mix which is as good as the commercially available ones. In this effort, our lab has optimized a beads mix for purifying and size selecting crude DNA extracted from eucalyptus and posted on
protocol.io, https://www.protocols.io/edit/high-purity-high-molecular-weight-dna-extraction-f-n5ydg7w?step=16.
However, this solution was not very effective in purifying crude DNA extracted from fungal material. DNA extracted from fungal material is highly viscous which is indicative of high levels of impurities in the DNA preparation. I tried to improve the beads mix for purifying rust DNA by adding 0.25 % (v/v) Tween-20 into the beads mix.
I tested beads mix with Tween-20 to see if adding tween into the solution makes any difference in the recovery, purity and size selection. Turns out that bead solutions with Tween-20 make big difference in the size selection and recovery of the DNA compared to the bead solutions without.

I calibrated/tested the bead solution with and without Tween-20 on the 1 kb DNA ladder to establish which DNA solution to beads volume ratio gives optimal recovery and size selection. We found that beads mix with 0.25 % Tween-20 works much better in size selection and recovery than beads mix without Tween-20. The best DNA to beads volume ratios were 1.0: 0.9 and 1.0: 1.0.

Citation: Ramawatar,Benjamin Schwessinger DNA size selection (>3-4kb) and purification of DNA using an improved homemade SPRI beads solution.. **protocols.io**
dx.doi.org/10.17504/protocols.io.n7hdhj6
Published: 09 Apr 2018

Guidelines

Beads protocol was originally adapted from Philippe Jolivet and Joseph W. Foley, 2015 - Solutions for purifying nucleic acids by solid-phase reversible immobilization (SPRI)
Link: http://www.openwetware.org/images/f/f8/SPRI_buffers_v2_2.pdf

Calibrating the newly prepared beads mix: Every time you prepare new a fresh beads mix, make sure you test it with either ladder DNA or DNA which you don't mind losing.

This is an open access protocol distributed under the terms of the Creative Commons Attribution License, which permits unrestricted use, distribution, and reproduction in any medium, provided the original author and source are credited

pH makes a huge difference in the solubility of DNA and beads. If not set properly, beads tend to clump at DNA elution step that could lose as much 60-70 percent of the DNA. So make sure that your elution buffer (e.g. 10 mM Tris or 0.1 x TE) has a pH of 8.

Homogeneous beads mix at room temperature before use.

Always use freshly prepared 70 % Ethanol
Preheat your elution buffer of choice (TE-Buffer, Tris 10 mM, Water..) to 37-42°.

We use same beads solution for the MinION sequencing library preparation.

Materials

- 0.5 M EDTA by Contributed by users
- 1 M Tris-HCl pH 8.0 by Contributed by users
- Ethanol 70% by Contributed by users
- 10 % Tween-20 by Contributed by users
- 10 mM Tris-HCL pH 8.0 by Contributed by users
- 50 % Polyethylene Glycol (PEG) by Contributed by users
- 5 M NaCl by Contributed by users

Protocol

Make beads stock solution

Step 1.

For 10 mL beads stock solution:

Final	stock	Input
10 mM Tris-HCl	1 M	100 µl
1 mM EDTA pH 8	0.5 M	20 µl

This is an open access protocol distributed under the terms of the Creative Commons Attribution License, which permits unrestricted use, distribution, and reproduction in any medium, provided the original author and source are credited

1.6 M NaCl	5 M	3.2 ml
11% PEG 8000	50% (w/v)	2.2 ml
0.25 % Tween-20	10 % (v/v)	200
0.4% beads (v/v)	100%	40 µl
Milliq Water		4.24 ml
Total		10 ml

Frist combine only Water, Tris-HCl, EDTA and NaCl in a 50 mL tube.

ANNOTATIONS

200(uL) of 10% Tween-20 stock in 10mL gives a final Tween-20 concentration of 0.2%. Is 0.25% or 0.2% correct?

Make beads stock solution

Step 2.

Vortex Sera-Mag SpeedBeads® Carboxyl Magnetic Beads (GE Healthcare) very well and pipette 40 µl into a 1.5 ml tube, put it on the magnetic rack and wait until the solution has cleared up and all beads have bound to the back of the tube

Make beads stock solution

Step 3.

Wash beads by removing supernatant and adding 1 ml Milli-Q water

Make beads stock solution

Step 4.

Take tube of the magnet, mix well, spin down in a microcentrifuge and put back on the magnet

Make beads stock solution

Step 5.

Wait for beads to assemble at the back of the tube

Make beads stock solution

Step 6.

Pipette off and discard supernatant

Make beads stock solution

Step 7.

Repeat washing (steps 3 - 6) 3 more times

This is an open access protocol distributed under the terms of the Creative Commons Attribution License, which permits unrestricted use, distribution, and reproduction in any medium, provided the original author and source are credited

Make beads stock solution

Step 8.

After pipetting of the supernatant the last time take off tube from the magnet and add 40 µl of the previous (step 1) prepared stock solution, mix well, spin down and pipette everything into the remaining stock solution in the 50 mL tube and mix

Make beads stock solution

Step 9.

Now the 2.2 ml 50% PEG can be added to the stock solution, which after vortexing very well is ready for use.

Be careful to actually pipette 2.2 ml as solution is very viscous, but the final concentration of PEG is crucial for the clean up to work properly.

Clean up

Step 10.

Bring you DNA sample in a 1.5 ml tube to comfortable pipetable volume (I usually do it with some volume between 20 - 200 µl) and if you know already that your sample contains a lot of contaminants and/or DNA (hence a really viscous solution) diluting it out and splitting into two tubes can make life easier.

(A lot of contaminants can also clump around the beads which makes binding to the magnet slower and sometimes pipetting off the supernatant really difficult)

Clean up

Step 11.

Make sure to know the exact volume of your sample and add 1 V of that (or 0.9 V for removal of more smaller fragments but also higher risk of less recovery) of well homogenized, room temperature beads solution and mix by flicking the tube

Clean up

Step 12.

Place the tube on a rotor (or mixer if you don't have one) and mix for 10 minutes

Clean up

Step 13.

Spin down tube in the microcentrifuge and place on the magnet

Clean up

This is an open access protocol distributed under the terms of the Creative Commons Attribution License, which permits unrestricted use, distribution, and reproduction in any medium, provided the original author and source are credited

Step 14.
Wait until beads have moved to the back of the tube and the solution becomes clear

(depending on viscosity of the solution that can take between 1 min to much longer like 1h, if after that the beads look like they are stuck in a big cloud and just don't properly bind to the magnet I add same amount of buffer and beads (the ratio always has to stay the same!) mix again and the put it back on the magnet - usually that solved the problem. But you probably will never have that problem, I just worked with really contaminated samples (plants))

Clean up

Step 15.
Remove and discard supernatant

Clean up

Step 16.
Wash beads with fresh 70% Ethanol by adding 1 - 1.5 mL to the opposite side than where the beads bind and wait 30 seconds

Clean up

Step 17.
Remove and discard Ethanol

Clean up

Step 18.
Repeat washing once more (steps 15 - 18)

(Don't remove tube from magnet during the washing steps until here)

Clean up

Step 19.
For the last removal of Ethanol make sure that all the Ethanol is removed, therefore take tube off the magnet , spin down for a second and place back onto the magnet, like that also the last drops of Ethanol can be pipetted off

Clean up

Step 20.
Let beads air dry for a maximum of 30 seconds or else elution will be difficult

Clean up

Step 21.
Add 50 ul (or in whatever final volume and concentration the sample is needed) of preheated to 50°C 10

This is an open access protocol distributed under the terms of the Creative Commons Attribution License, which permits unrestricted use, distribution, and reproduction in any medium, provided the original author and source are credited

mM Tris (or TE-Buffer)

Clean up

Step 22.

Make sure the beads are resuspend properly by flicking the tube gently and spinning it down - the solution will be homogeneous and brown

(I put it in a 50°C Thermoblock for about 20 seconds to encourage the elution reaction)

Clean up

Step 23.

Spin down the tube before placing it on the magnet again and wait until the beads have bound the the back of the tube (that can again take its time especially if the sample contains really long DNA fragments) Depending on the sample that will take between 1 minute to a few hours, I usually wait 5 - 10 minutes

Clean up

Step 24.

When the solution has cleared up completely, pipette the supernatant to a fresh tube and discard beads.

Quality control

Step 25.

Meassure the DNA concentration with a Qubit.
The recoveries for HMW DNA > 20kb should lie between 60 - 90%

Quality control

Step 26.

Run a 1.0% TBE agarose gel

80 ng DNA per sample, normalized to 10 ul per lane

1 2 3 4 5 6 7 8 9 10 11

This is an open access protocol distributed under the terms of the Creative Commons Attribution License, which permits unrestricted use, distribution, and reproduction in any medium, provided the original author and source are credited

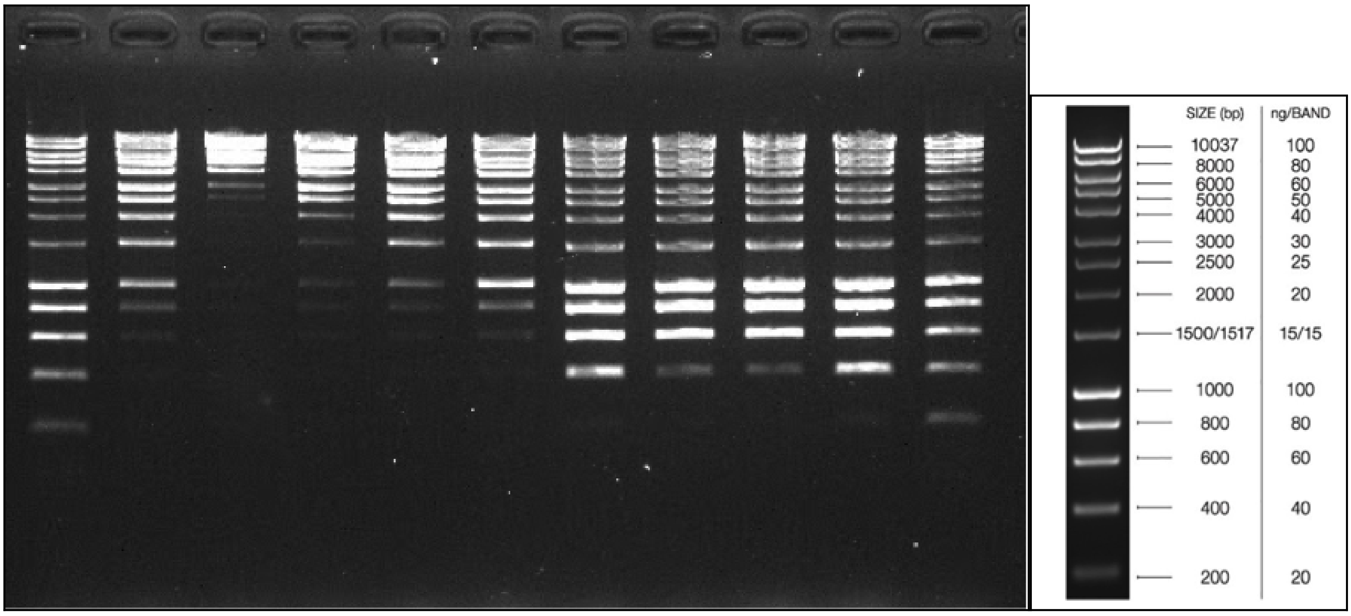

Lanes:
Lane 1 and 11: Bioline 1kb hyperladder

Lane 2:0.45 AMPure XP Beads (Beckman Coulter). This is the standard dilution used in PacBio and Oxford Nanopore protocols.

Lane 3: 1.0:0.8 V our SPRI beads with Tween-20
Lane 4: 1.0:0.8 V our SPRI beads w/o Tween-20
Lane 5: 1.0:1.0 V our SPRI beads with Tween-20
Lane 6: 1.0:1.0 V our SPRI beads w/o Tween-20
Lane 7: 1.0:1.5 V our SPRI beads with Tween-20
Lane 8: 1.0:1.5 V our SPRI beads w/o Tween-20
Lane 9: 1.0:2.0 V our PRI beads with Tween-20
Lane 10: 1.0:2.0 V our SPRI beads w/o Tween-20

This is an open access protocol distributed under the terms of the Creative Commons Attribution License, which permits unrestricted use, distribution, and reproduction in any medium, provided the original author and source are credited
